# Spatiotemporal spread of sarcoptic mange in the red fox (*Vulpes vulpes*) in Switzerland over more than 60 years: lessons learnt from comparative analysis of multiple surveillance tools

**DOI:** 10.1186/s13071-019-3762-7

**Published:** 2019-11-05

**Authors:** Simone Roberto Rolando Pisano, Fridolin Zimmermann, Luca Rossi, Simon Capt, Ezgi Akdesir, Roland Bürki, Florin Kunz, Francesco Carlo Origgi, Marie-Pierre Ryser-Degiorgis

**Affiliations:** 1 Centre for Fish and Wildlife Health (FIWI), Department of Infectious Diseases and Pathobiology, Vetsuisse Faculty, University of Bern, Laenggassstrasse 122, PO Box, 3001 Bern, Switzerland; 2 KORA – Carnivore Ecology and Wildlife Management, Thunstrasse 31, 3074 Muri, Switzerland; 3 Dipartimento di Scienze Veterinarie, Università degli Studi di Torino, Largo Braccini 2, 10095 Grugliasco, Italy; 4 Info Fauna, Swiss Centre for the Cartography of the Fauna, Bellevaux 51, 2000 Neuchâtel, Switzerland; 5 Swiss Rabies Centre, Institute of Virology and Immunology (IVI), Vetsuisse Faculty, University of Bern, Laenggassstrasse 122, PO Box, 3001 Bern, Switzerland

**Keywords:** *Sarcoptes scabiei*, Scabies, Rabies, Camera-trapping, Questionnaire, Necropsy, Post-mortem examination, Surveillance, Disease interference

## Abstract

**Background:**

Sarcoptic mange is a contagious skin disease of wild and domestic mammals caused by the mite *Sarcoptes scabiei*. Reports of sarcoptic mange in wildlife increased worldwide in the second half of the 20th century, especially since the 1990s. The aim of this study was to provide new insights into the epidemiology of mange by (i) documenting the emergence of sarcoptic mange in the red fox (*Vulpes vulpes*) in the last decades in Switzerland; and (ii) describing its spatiotemporal spread combining data obtained through different surveillance methods.

**Methods:**

Retrospective analysis of archived material together with prospective data collection delivered a large dataset from the 19th century to 2018. Methods included: (i) a review of historical literature; (ii) screening of necropsy reports from general health surveillance (1958–2018); (iii) screening of data on mange (1968–1992) collected during the sylvatic rabies eradication campaign; (iv) a questionnaire survey (<1980–2017) and (v) evaluation of camera-trap bycatch data (2005–2018).

**Results:**

Sarcoptic mange in red foxes was reported as early as 1835 in Switzerland. The first case diagnosed in the framework of the general health surveillance was in 1959. Prior to 1980, sarcoptic mange occurred in non-adjacent surveillance districts scattered all over the country. During the period of the rabies epidemic (1970s-early 1990s), the percentage of foxes tested for rabies with sarcoptic mange significantly decreased in subregions with rabies, whereas it remained high in the few rabies-free subregions. Sarcoptic mange re-emerged in the mid-1990s and continuously spread during the 2000–2010s, to finally extend to the whole country in 2017. The yearly prevalence of mange in foxes estimated by camera-trapping ranged from 0.1–12%.

**Conclusions:**

Sarcoptic mange has likely been endemic in Switzerland as well as in other European countries at least since the mid-19th century. The rabies epidemics seem to have influenced the pattern of spread of mange in several locations, revealing an interesting example of disease interaction in free-ranging wildlife populations. The combination of multiple surveillance tools to study the long-term dynamics of sarcoptic mange in red foxes in Switzerland proved to be a successful strategy, which underlined the usefulness of questionnaire surveys.

## Background

The burrowing mite *Sarcoptes scabiei* (Acarina: Sarcoptidae) is the aetiological agent of sarcoptic mange, a highly contagious skin disease affecting more than a hundred of domestic and wild mammal species worldwide [1, 2]. Susceptibility to sarcoptic mange and the resulting clinical picture, pathological features and mortality vary substantially between species [1, 3, 4]. Among native and invasive European wild carnivores, sarcoptic mange has been reported in canids, felids, mustelids and procyonids [5–9].

In free-ranging populations sarcoptic mange can have either an epidemic or an endemic nature, with an initial epidemic occurrence gradually transitioning to an endemic one [10]. Epidemics of sarcoptic mange rapidly spread throughout naïve populations over broad geographical areas and are associated with dramatic drops in host density and substantial structural shifts in population dynamics [7, 11–15]. Impressive epidemics of sarcoptic mange in red foxes (*Vulpes vulpes*; from here simply referred to as “fox(es)”) and other wild carnivores occurred in Fennoscandia and Denmark in the 1960s–1990s [7, 14, 16], where regional fox mortality reached 90% [7, 17]. Several outbreaks in foxes were also reported in Great Britain in the 1990s [13].

Endemic situations are characterized by a cyclic pattern. Outbreaks are typically limited in time and space and followed by apparently long disease absences and a parallel emergence in adjacent regions [18, 19]. Mortality is generally low, implying only short-term local population decreases [1, 18, 20]. Endemic situations in fox populations have been described in several European countries, such as Denmark [21], Germany [20], Poland [22], Spain [18] and Great Britain [13, 23].

Although known since biblical times in both humans (disease referred to as scabies) and livestock [24, 25], long-term documentation of sarcoptic mange in wildlife populations is rare [1] and often found in non-English literature only. Early scientific descriptions of sarcoptic mange in wildlife date back to the end of the 19th century and the first half of the 20th century [26–30] and *S. scabiei* in foxes has been documented since the beginning of the 19th century [31]. Nevertheless, reports of sarcoptic mange in wild mammalian species, including foxes and other sympatric carnivores in Europe have increased since the second half of the 20th century, becoming exponential since the 1990s [7, 13, 15, 32–37]. This increase was interpreted as an emerging disease new to some parts of Great Britain [38, 39] and Germany [40]. The reasons for this trend are still unknown and may be attributable to changes in the interactions among mites, hosts and their environment, as well as to the increase of wildlife disease surveillance and research.

In Switzerland, epidemics of sarcoptic mange in foxes were reported in the Northwestern Alps and around the city of Geneva between 1996 and 1998 [11, 41]. The disease was also detected in mustelids [6] and in the reintroduced Eurasian lynx (*Lynx lynx*) population for the first time in 1999 [5]. Since then, sarcoptic mange has also been reported in wild boar (*Sus scrofa*), although in this species it is likely caused by different *S. scabiei* strain(s) [42]. These findings raised the question as whether mange was an emerging disease new to Switzerland, whether it would further spread and which impact it would have on autochthonous free-ranging wildlife.

The aim of this study was to provide new insights into the epidemiology of sarcoptic mange by (i) documenting the emergence of sarcoptic mange in the red fox in the last decades in Switzerland; and (ii) describing its spatiotemporal spread by combining data obtained with different surveillance methods.

## Methods

### Study area

**Fig. 1:**
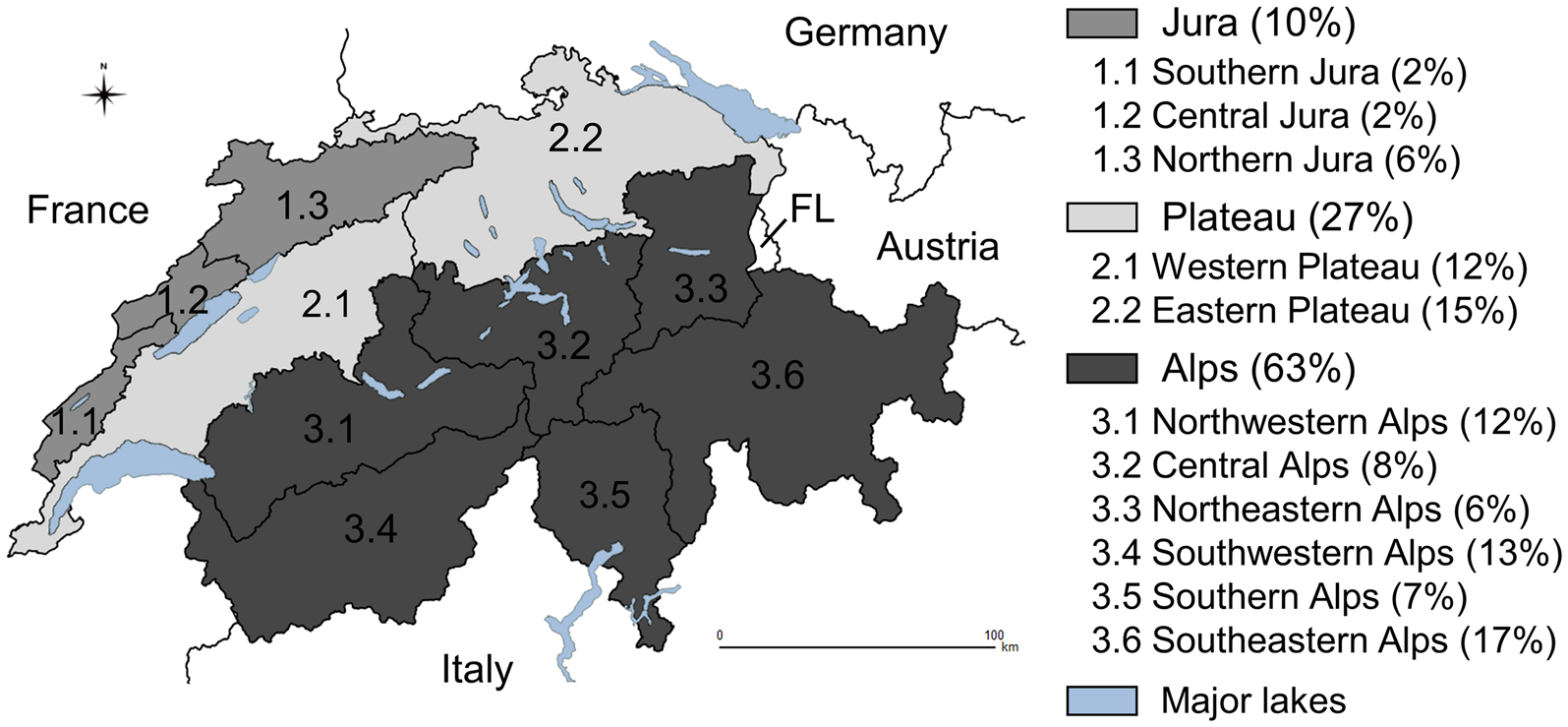
Biogeographical regions and subregions of Switzerland. Shades of grey refer to the three biogeographical regions. Black lines border the eleven biogeographical subregions. The percentage of country surface area covered by the regions and subregions is given in brackets. Neighbouring countries are indicated with their names. *Abbreviation*: FL, Principality of Liechtenstein

Switzerland (41,285 km^2^) is an Alpine country in central Europe. It is divided into 26 political subunits (cantons) further subdivided in districts of surveillance of different sizes belonging to three main biogeographical regions (Alps, Plateau and Jura, covering around 63%, 27% and 10% of the country surface area (CSA), respectively) and 11 subregions (with the respective cardinal and intercardinal points spelled with capital letters, Fig. 1).

### Case definition

For this study, mange-like lesions (MLL) were defined as mild to severe encrustations on the skin with or without alopecia, skin thickening and hyperpigmentation, characterized by a typical body distribution and progression of the lesions [4]. Sarcoptic mange was defined as the presence of MLL associated with the detection of intradermal mites consistent with *S. scabiei*. MLL (with or without mite detection) are typical signs of subacute to chronic stages of *S. scabiei* infection and can therefore be used for disease detection.

### Study design

Data obtained through the following five methods were combined: (i) a review of historical literature; (ii) screening of necropsy reports from general health surveillance (1958–2018); (iii) compilation of data on sarcoptic mange (1968–1992) collected during the sylvatic rabies eradication campaign; (iv) a questionnaire survey (<1980–2017); and (v) evaluation of camera-trap bycatch data (2005–2018).

#### Review of the historical literature

The aim of the literature review was to discover the oldest mention of MLL in foxes in Switzerland and in neighbouring countries. The following online databases were consulted: PubMed (http://www.ncbi.nlm.nih.gov/pubmed/), Google Books (http://www.books.google.com) Google Scholar (http://www.scholar.google.com), Zobodat (http://www.zobodat.at), Gallica (http://www.gallica.bnf.fr), E-periodica (http://www.e-periodica.ch), E-rara (http://www.e-rara.ch), E-manuscripta (http://www.e-manuscripta.ch), the Swiss National Library (http://www.nb.admin.ch) and the Library of the Universities of Bern and Basel (http://www.baselbern.swissbib.ch). The following keywords were used in English, German, French and Italian: (i) sarcoptic mange (*Sarcoptes scabiei*, scabies, mange, itch, mite); (ii) red fox (*Vulpes vulpes*, fox); and (iii) Switzerland, Austria, Italy, France, Germany and Principality of Liechtenstein.

#### General health surveillance

In total, 1128 archived necropsy reports of foxes examined at the Centre for Fish and Wildlife Health (FIWI) between 1958 and 2018 (except for 1982: missing files) were screened for MLL and sarcoptic mange. The coordinates of origin were known for 1120 foxes (99%). Based on the information included in the reports, animals were classified as juveniles (< 6 months-old) or subadults/adults (≥ 6 months-old), assuming the 1st of April as the date of birth of fox cubs [43]. Cases were placed in two 6-months periods (colder months: October–March and warmer months: April–September) according to their submission date.

**Fig. 2:**
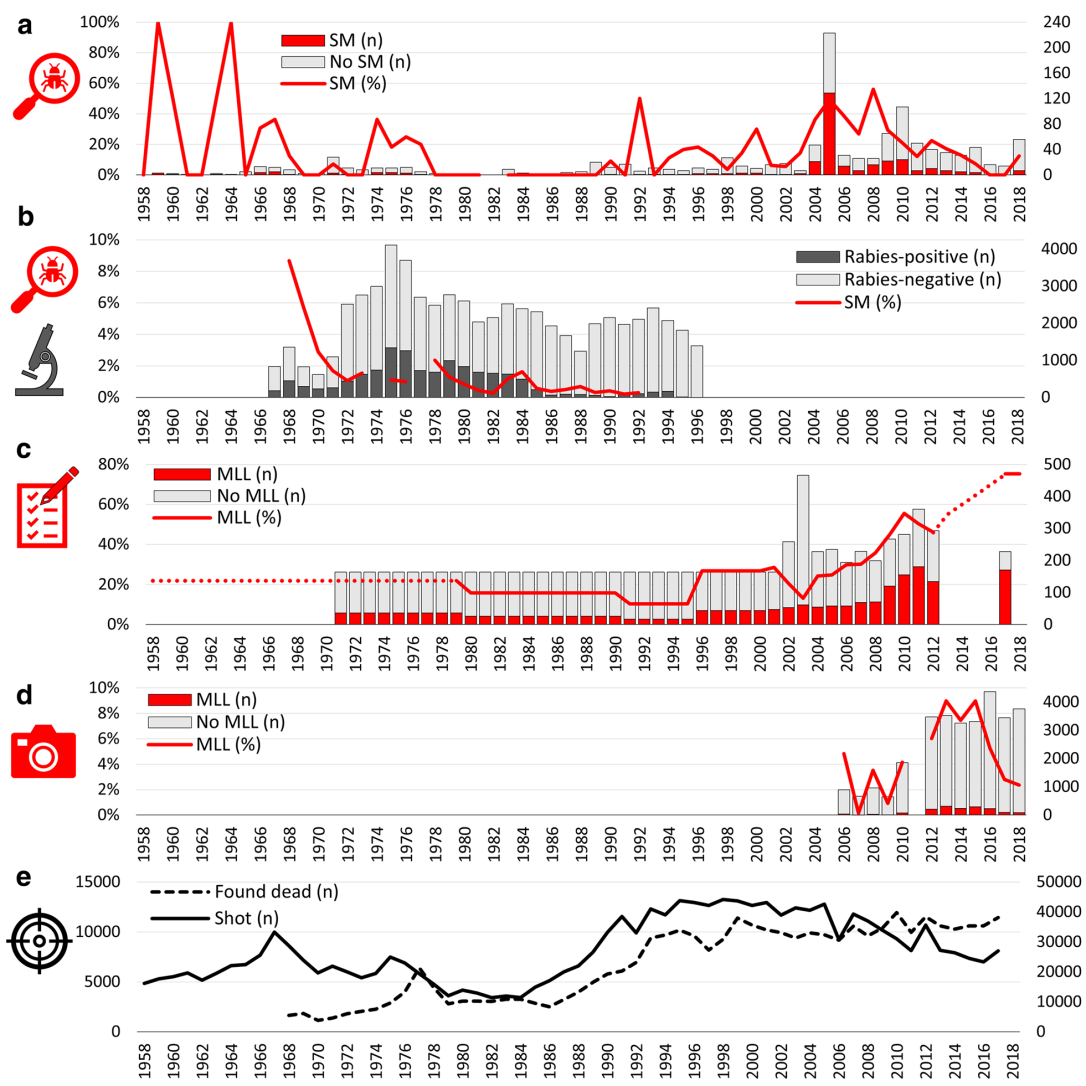
Temporal occurrence of sarcoptic mange cases in Switzerland according to the surveillance method (1958–2018). **a** Percentage of sarcoptic mange and number of red foxes (*Vulpes vulpes*) with and without sarcoptic mange (confirmed through mite identification) submitted in the framework of the general health surveillance programme for wildlife (source: FIWI). **b** Percentage of foxes with sarcoptic mange (1968–1992; data not available for 1974 and 1977) and number of foxes analysed and tested for rabies (1967–1996) during the rabies eradication campaign (source: SRC). **c** Percentage and number of questionnaires reporting mange-like lesions (not confirmed by mite identification) in the framework of a yearly questionnaire survey (2001–2017; source: FIWI). **d** Number and prevalence of camera-trap pictures showing foxes with mange-like lesions. Pictures were bycatch material collected during the Eurasian lynx camera-trapping monitoring (2005–2018; source: KORA). **e** Number of foxes culled or found dead at national level (source: national hunting statistics). The left y-axes of diagrams **a**-**d** represents the respective percentages or prevalences. The left y-axis of diagram **e** represents the number of foxes shot. The right y-axes represent the number of units analysed and differ among data sources (**a** necropsies performed, **b** foxes examined for rabies, **c** questionnaires returned, **d** camera-trap pictures, **e** foxes found dead). *Abbreviations*: *MLL*, mange-like lesions; *SM*, sarcoptic mange

Carcasses and organs of foxes of both sexes (558 males, 49%; 505 females, 45%; 65 of unknown sex, 6%) and age classes (140 juveniles, 12%; 933 subadults/adults, 83%; 55 of unknown age, 5%) were submitted from all biogeographical subregions, mostly from the Western Plateau (22%), the Eastern Plateau (20%) and the Northwestern Alps (19%). A total of 694 foxes (62%) had been culled, while 399 (35%) animals were found dead. In 35 cases (3%) there was no additional information about whether foxes had been found dead or culled. The number of performed necropsies (Fig. 2a) showed a similar pattern as the national hunting statistics (Fig. 2e; https://www.uzh.ch/wild/static/jagdstatistik/).

The method used for confirming *S. scabiei* infestation varied during the study period. From 1958 to 2003, deep dry skin scraping was used to isolate mites. Since 2004, isolation of mites has been attempted through a heating stimulation technique (i.e. skin samples placed in Petri dishes under a light source) and/or deep dry skin scraping [4, 44]. Identification of *S. scabiei* was performed through morphological characterization using a light microscope [45]. If isolation failed, the presence of intradermal mites was investigated through histopathology [46].

Carcass collection intensity and the institutions involved varied throughout the study period: during the rabies epidemic peak (1970–1980s [47]), foxes were sent to the Swiss Rabies Centre (SRC) rather than to the FIWI. From 2002 to 2013, questionnaire participants (see below) were reminded each year to submit foxes with MLL to the FIWI for post-mortem examination to validate the data obtained through the questionnaire survey. In 2004–2005, foxes with MLL were actively collected for a study on sarcoptic mange [4]. Since 2009, an increased number of foxes have been submitted due to epidemic waves of canine distemper [48, 49].

#### Data on mange collected during the sylvatic rabies eradication campaign

**Fig. 3:**
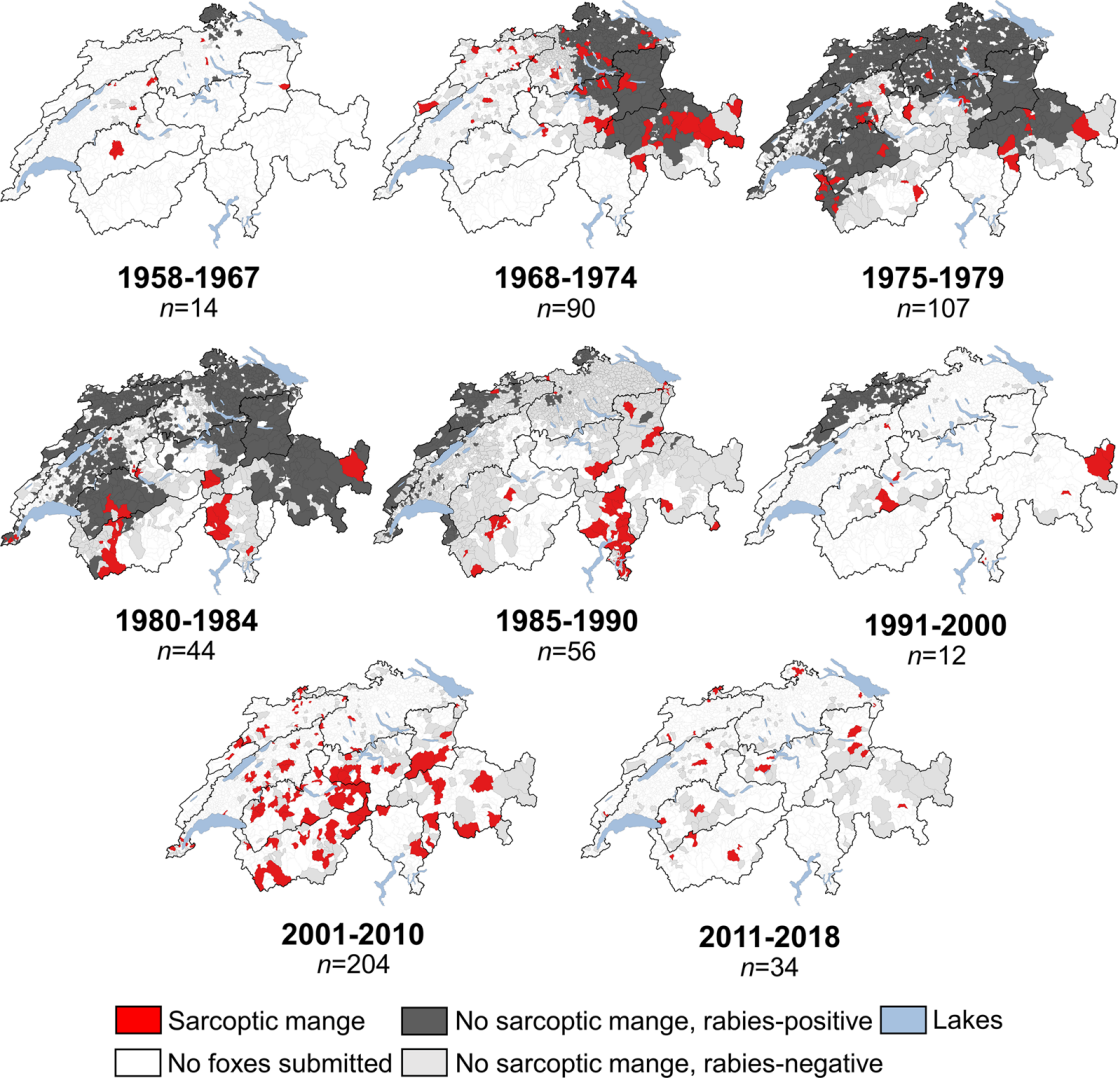
Spatiotemporal distribution of red foxes with sarcoptic mange in Switzerland (1958–2018). Municipalities from where red foxes (*Vulpes vulpes*) were submitted in the framework of the general health surveillance programme for wildlife (1958–2018; source: FIWI) and during the initial part of the rabies eradication campaign (1968–1990; data on mange not available for 1974 and 1977; source: SRC) are illustrated with different colours depending on whether foxes had sarcoptic mange or not and whether they were tested positive or negative for rabies. Sarcoptic mange was defined as the presence of mange-like lesions confirmed by the detection of *Sarcoptes scabiei*. In the periods 1958–1967, 1991–2000, 2001–2010, 2011–2018 only the municipalities with rabies occurrence (data on mange not available; source: SRC) and those without rabies analysed at the FIWI (data on mange available) were illustrated. The total number of foxes with sarcoptic mange is indicated below the corresponding period

Archival data on sarcoptic mange (MLL with confirmed *S. scabiei* infestation) collected on foxes tested for rabies at the SRC from 1968 to 1992 (except for 1974 and 1977: missing reports on mange) were screened (*n* = 29,241; database of the SRC). Coordinates of mangy foxes were only available for foxes submitted from 1968 to 1990. Mite isolation was attempted through deep skin scraping and microscopic characterization. The number and geographical origin of foxes tested positive for rabies from 1967 to 1996 (last case of rabies in a fox [50]) were illustrated to help the interpretation of the data on sarcoptic mange (Figs. 2b, 3; database of the SRC [47, 51]).

#### Questionnaire survey

A questionnaire was sent to professional game wardens and hunters responsible for a district of surveillance (i.e. wildlife management subunits) every year from 2002 to 2013 and again in 2018 (12 survey rounds in total). The questionnaire gathered information about the observation of foxes with MLL in the respective districts during the previous year (i.e. 2001–2012 and 2017) and consisted in four closed questions (Table 1). In the first survey round (2002) an additional question was included on MLL occurrence in previous decades (before 1980, 1980–1990, 1991–1995, 1996–2000).

**Table 1:** Questions (and possible answers) of the yearly questionnaire survey on sarcoptic mange in red foxes in Switzerland

Questions		Answers				
1	Did you observe foxes with MLL?	Yes	No	–	–	–
2	How many foxes were affected?	None	1–2	3–5	6–10	> 10
3	If you observed cases, which type(s) of observation did you make?	Live fox(es) with MLL	Fox(es) found dead with MLL	Culled fox(es) with MLL	Mite detection in a laboratory	–
4	How do you consider the trend of MLL occurrence	Increasing	Stable	Decreasing	–	–

*Abbreviation*: MLL, mange-like lesions

Questionnaires were filled either by the cantonal hunting office for the entire canton or by the persons in charge of the single districts, resulting in different numbers of returned questionnaires per canton and per year. In total, 4054 exploitable questionnaires were returned to the FIWI. The answers were aggregated in nine time periods (before 1980, 1980–1990, 1990–1995, 1996–2000, 2001–2003, 2004–2006, 2007–2009, 2010–2012 and 2017). The lowest number of foxes with MLL reported yearly as a range was calculated by adding the lowest value of the answers of the second question of the questionnaire (i.e. how many foxes were affected, indicated as a range). The mean of these values was calculated for each period. MLL occurrence was expressed as the percentage of responding cantons and districts reporting MLL and as the percentage of CSA covered by districts reporting MLL, in an attempt to quantify the spatial spread of sarcoptic mange.

#### Camera-trapping

Pictures of foxes collected as bycatch data in the framework of a long-term camera-trap monitoring programme for the Eurasian lynx conducted by KORA (Carnivore Ecology and Wildlife Management) were assessed for the presence of MLL in foxes. The monitoring was standardized and performed during 23 sessions. Each session generated data recorded during subsequent 60 days between 2005 and 2018 in seven sectors belonging to six subregions (Additional file 1: Table S1) using Xenon white flash camera-traps with a fast trigger speed to produce high quality pictures [52, 53]. Because each session was performed in winter, either from December to February (phase 1) or from February to April (phase 2), the year used to designate a session corresponded to the year when the session ended (Additional file 1: Table S1). In total, 69,116 pictures were obtained and evaluated.

In a first step, all pictures of foxes (*n* = 30,168, 44%) were screened for the presence of MLL by KORA collaborators. In a second step, 7421 of these pictures were randomly selected and re-assessed for MLL by a wildlife veterinarian (first author); the original assessment was masked to ensure an objective re-evaluation. Cohen’s kappa was computed to assess inter-observer agreement between the two evaluations [54]. According to the Cohen’s kappa test value (Cohen’s kappa: *k* = 0.8; *Z* = 68.6; *P* < 0.0001; see Data analyses section below), the KORA evaluation was considered sufficient for the calculation of the MLL prevalence using all fox pictures of the database. For each session, MLL prevalence was estimated at picture level: number of pictures of foxes with MLL/total number of fox pictures.

#### Hunting statistics

The national and cantonal hunting bags and the number of foxes found dead from 1958 to 2017 were obtained from the free-access website of the national hunting statistics: https://www.uzh.ch/wild/static/jagdstatistik/. Normalized hunting bags in relation to the preferred habitat of foxes (HIPD: hunting indicator of fox population density, i.e. number of foxes shot/terrestrial surface (km^2^) below 2000 meters of altitude [55]) were used as an index of fox density in the respective areas [56]. The cantonal HIPDs during four 4-year periods (before, 1946–1950; during, 1980–1984; and after the rabies epidemics, 1993–1997 and 2013–2017), the respective factor of increase (e.g. HIPD_80–84_/HIPD_46–50_) and rate of increase, e.g. (HIPD_80–84_ −HIPD_46–50_)/(34 years × 100) were calculated according to Müller et al. [56] and used to help the interpretation of the sarcoptic mange trends. These data have already been used in other studies to detect the effect of rabies on fox population in Switzerland [43, 56].

### Data analyses

Data management and descriptive statistics were conducted in Microsoft Excel and Access 2016 (Microsoft Corporation, Redmond, Washington, USA). Statistical analyses were performed with R version 3.4.3 (http://www.r-project.org). The *prevalence* package was used to calculate prevalence with a Clopper-Pearson confidence interval (CI) of 95% (propCI function). Prevalences were calculated only for the camera-trapping data because the reference population was the entire fox population living in the assessed sector (in contrast to the collection of diseased foxes selected by field partners). Cohen’s kappa was computed using the *irr* package [54]. The exact binomial test (binom.test function) was used to compare sex and age of fox carcasses submitted to the FIWI. The test of equal or given proportions (TEP, prop.test function) and the pairwise comparisons for proportions with Holm–Bonferroni adjustment method (pairwise.prop.test function) were computed to compare percentages and prevalences. The level of significance was set at *P* < 0.05. Maps were designed using qGIS version 2.18.5 (http://www.qgis.org). The percentages of CSA covered by districts or municipalities with or without mange occurrence were obtained in qGIS.

## Results

### Review of the historical literature

The oldest document mentioning mange in foxes in Switzerland was a book on fox diseases published in 1835 [57]. In neighbouring countries, mange in foxes was mentioned in a human medical book published in Italy in 1560 [58], in a French forensic medicine and public health book from 1813 [59], in a German natural history newspaper from 1772 [60] and in a natural history book published in Austria in 1855 [61]. Although no historical information on mange for the Principality of Liechtenstein was found, sarcoptic mange has been present in the fox population of this country at least since 2012 (FIWI archives, unpublished observation).

### General health surveillance

The oldest case of confirmed sarcoptic mange dates back to 1959 and originated from the Northwestern Alps. MLL were detected in 326 (29%) foxes (51% males; 45% females; 4% unknown sex). Intradermal mites consistent with *S. scabiei* were found in 278 (85%) foxes with MLL. The percentage of foxes with confirmed sarcoptic mange did not significantly differ between males (26%) and females (24%, TEP: *χ*^2^ = 37844, *df* = 1, *P* = 0.5384), while subadults/adults (27%) were significantly more affected than juveniles (11%; TEP: *χ*^2^ = 15.653, *df* = 1, *P* < 0.0001). The percentage of MLL was significantly higher in foxes that were culled (35%) than in foxes found dead (20%; TEP: *χ*^2^ = 26.546, *df* = 1, *P* < 0.0001) and also higher in foxes that were submitted during the colder semester (34%) than during the warmer semester (21%; TEP: *χ*^2^ = 23.075, *df* = 1, *P* < 0.0001). The monthly percentage of foxes with MLL is shown in Additional file 2: Figure S1. The highest MLL percentage was detected in December (57%), the lowest MLL percentage in June (10%).

The percentage and geographical distribution of foxes with MLL (with or without mite detection) varied over the entire study period (Table 2, Figs. 2a, 3; Additional file 2: Tables S3 and S4, Figures S2–S4). Foxes with confirmed sarcoptic mange were mostly submitted from the Northwestern Alps (33%), Western Plateau (17%) and Southwestern Alps (16%; Fig. 3, Additional file 2: Figure S4).

**Table 2:** Spatiotemporal distribution of foxes with mange-like lesions from 1958 to 2018. Affected foxes (*Vulpes vulpes*) with mange-like lesions submitted from three biogeographical regions and eleven subregions in the framework of the general health surveillance programme for wildlife in Switzerland. Percentage (%) followed by the number of foxes affected/examined in parentheses

(Sub)region		1958–1969	1970–1979	1980–1989	1990–1999	2000–2009	2010–2018	Total
1	Jura	40 (2/5)	14 (2/14)	0 (0/9)	0 (0/15)	33 (13/39)	38 (14/37)	26 (31/119)
1.1	Southern Jura	nd	nd	nd	nd	nd	50 (1/2)	50 (1/2)
1.2	Central Jura	nd	nd	0 (0/1)	0 (0/1)	50 (2/4)	0 (0/1)	29 (2/7)
1.3	Northern Jura	40 (2/5)	14 (2/14)	0 (0/8)	0 (0/14)	31 (11/35)	38 (13/34)	25 (28/110)
2	Plateau	31 (9/29)	22 (6/27)	0 (0/13)	5 (3/56)	24 (36/153)	15 (28/181)	18 (83/459)
2.1	Western Plateau	33 (6/18)	24 (6/25)	0 (0/9)	3 (1/29)	28 (31/110)	14 (7/49)	22 (51/240)
2.2	Eastern Plateau	27 (3/11)	0 (0/2)	0 (0/4)	7 (20/27)	12 (5/43)	16 (21/132)	22 (49/219)
3	Alps	67 (6/9)	18 (8/45)	0 (0/13)	28 (11/40)	61 (167/276)	13 (21/159)	39 (214/542)
3.1	Northwestern Alps	63 (5/8)	29 (2/7)	0 (0/10)	22 (6/27)	71 (88/124)	8 (3/36)	49 (104/212)
3.2	Central Alps	nd	nd	0 (0/1)	nd	55 (12/22)	17 (5/30)	32 (17/53)
3.3	Northeastern Alps	100 (1/1)	nd	0 (0/1)	0 (0/2)	7 (1/15)	39 (7/18)	24 (9/37)
3.4	Southwestern Alps	nd	nd	nd	0.0 (0/1)	65 (50/77)	24 (4/17)	57 (54/95)
3.5	Southern Alps	nd	nd	0 (0/1)	67 (2/3)	83 (5/6)	0 (0/3)	54 (7/13)
3.6	Southeastern Alps	nd	16 (6/38)	nd	43 (3/7)	34 (11/32)	4 (2/55)	17 (22/132)
Switzerland		40 (17/43)	19 (16/86)	0 (0/35)	13 (14/111)	46 (216/468)	17 (63/377)	30 (326/1120)

*Abbreviation*: nd: no data

### Data on mange collected during the rabies eradication campaign

Sarcoptic mange was reported in 306 (1%) foxes. Mangy foxes were submitted from all subregions but mainly from the Southern Alps (31%), the Eastern Plateau (15%) and the Southwestern Alps (13%), and only rarely from the Jura (Fig. 3, Additional file 3: Table S5).

The percentage and distribution of foxes with sarcoptic mange varied with the occurrence and spread of rabies. The percentage rapidly decreased as the cumulative percentage of CSA with rabies occurrence increased, decreasing in the biogeographical subregions where rabies occurred, showing a similar pattern as the national hunting statistics (Figs. 2b, 3, Additional file 3: Figures S5–S7). More precisely, the percentage of sarcoptic mange was higher in the Southern (29%) and Southwestern (4%) Alps, where rabies only minimally spread due to the successful vaccination campaigns or had never occurred, respectively (Fig. 3, Additional file 3: Table S5, Figure S6) [51]. The percentage of sarcoptic mange significantly decreased from 1% (1968–1984) to 0.2% (1985–1992; TEP: *χ*^2^ = 114.06, *df* = 1, *P* < 0.0001) in all subregions except the Southern Alps, where the percentage (25%) was significantly higher during the same period (1985–1992; TEP: *χ*^2^ = 2118, *df* = 1, *P* < 0.0001; Additional file 3: Figure S7).

### Questionnaire survey

In total, MLL were reported in 1358 (34%) returned questionnaires. Detailed information on the results of the questionnaire survey are summarized in Additional file 4: Tables S6 and S7.

**Fig. 4:**
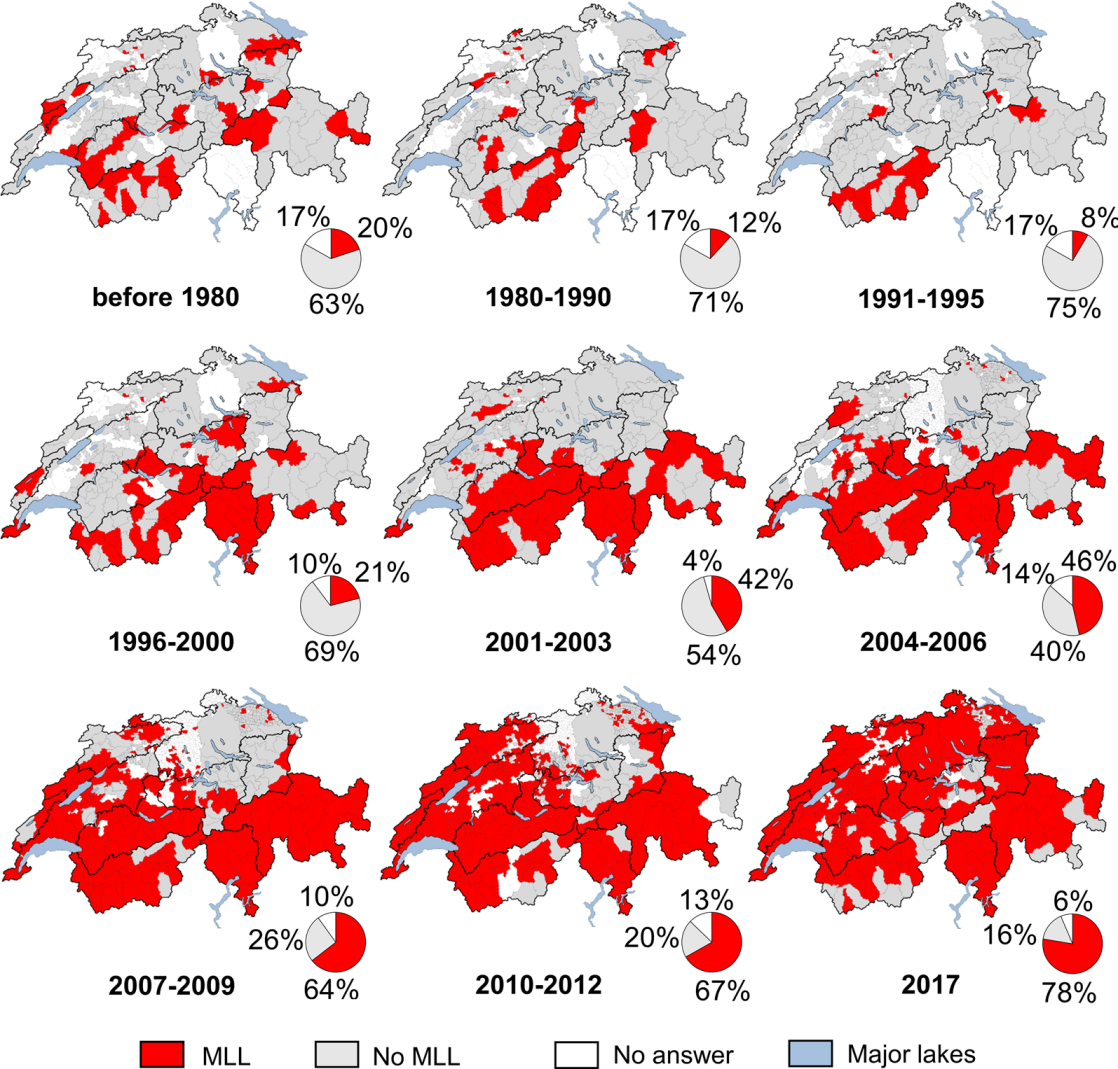
Spatiotemporal distribution of red foxes with mange-like lesions based on a multi-year questionnaire survey. The maps illustrate the districts of surveillance in Switzerland reporting foxes (*Vulpes vulpes*) with and without mange-like lesions per period. Pie charts represent the total country surface area of Switzerland and show the percentage of the total area corresponding to districts with reported occurrence or absence of mange-like lesions in foxes, or which did not reply to the questionnaire survey. Biogeographical subregions are delimited by black lines. *Abbreviation*: MLL, mange-like lesions

Before 1980, foxes with MLL were reported only in a few isolated, non-adjacent districts in all investigated subregions (20% CSA) (Table 3, Fig. 4, Additional file 4: Figures S8 and S9). The reports of foxes with MLL dropped from 1980 to 1995 and originated mainly from the Southwestern Alps (9–12% CSA), while only a few isolated reports came from the remaining Alpine subregions, from the Western Plateau and the Northern Jura (Table 3, Fig. 4, Additional file 4: Figures S8 and S9). From 1996, the number of reports increased again (21% CSA) and foxes with MLL were reported mainly from the Southwestern and Southern Alps and less frequently from the Northwestern and Central Alps (Table 3, Fig. 4, Additional file 4: Figures S8 and S9).

**Table 3:** Occurrence of foxes with mange-like lesions based on a multi-year questionnaire survey in Switzerland. Percentage (%) followed, in parentheses, by the number of districts of surveillance, cantons and subregions reporting red foxes (*Vulpes vulpes*) with mange-like lesions/number of responding districts of surveillance, cantons and subregions

	< 1980	1980–1990	1991–1995	1996–2000	2001–2003	2004–2006	2007–2009	2010–2012	2017
Districts of surveillance	22 (36/162)	16 (26/162)	10 (17/162)	27 (44/162)	18 (157/884)	26 (169/653)	37 (258/691)	50 (471/933)	75 (171/227)
Cantons	58 (14/24)	33 (8/24)	17 (4/24)	50 (12/24)	50 (13/26)	58 (14/24)	68 (17/25)	91 (21/23)	96 (25/26)
Subregions	100 (10/10)	80 (8/10)	60 (6/10)	81 (9/11)	72 (8/11)	81 (9/11)	100 (11/11)	100 (11/11)	100 (11/11)

From 2001 to 2017, the number and percentage of responding cantons and districts of surveillance reporting foxes with MLL, the corresponding CSA percentage and the total number of foxes with MLL continuously increased (Table 3, Fig. 4, Additional file 4: Figure S8 and S9). In 2001–2003, the distribution pattern was similar to that of the previous period (1996–2000), but the mange front moved slightly further north: the number of districts with mange occurrence increased both in the Northwestern and Southwestern Alps and in the Western Plateau (Fig. 4). In the Northern Jura, there was a slight increase of districts with only a few observations of foxes with MLL (Fig. 4, Additional file 4: Figure S10). In 2004–2006, MLL were again mainly reported from the southern half of the country, but the spread slowly continued towards north, especially in the Western Plateau (Fig. 4). Sarcoptic mange was confirmed and further spread in the Northern Jura (Fig. 4, Additional file 4: Figure S10). The situation in the Northeastern and Eastern Alps and in the Jura remained unchanged, with only a few unconfirmed reports (Fig. 4, Additional file 4: Figure S10). In 2007–2009, mange spread further north in the western part of Switzerland and further south in the eastern part of Switzerland. Since 2008, the Northwestern, Southwestern and Southern Alps, most of the Western Plateau, Southern Jura, Central Jura and Southeastern Alps have been entirely affected (Fig. 4). An increase of observations also came from the Northern Jura and cases were reported from the Northeastern Alps for the first time since the 1991–1995 period (Fig. 4, Additional file 4: Figure S10). On the contrary, in the Central Alps the spread was less pronounced, and the Eastern Plateau was still largely free of mange (Fig. 4). In 2010–2012, mange spread further in the northwestern and northeastern parts of Switzerland and MLL were reported from all of Switzerland except for the central part of the Eastern Plateau, the western part of the Eastern Alps and most of the Central Alps (Fig. 4). In 2017, the spread over the country was nearly complete: most of the districts reported MLL, especially in the Jura and in the Plateau (Fig. 4, Additional file 4: Figure S10). At the same time, an increased number of districts (mainly in the Alps) did not report foxes with MLL anymore, suggesting a local fading out of the epidemic (Fig. 4, Additional file 4: Figure S11).

The apparent discrepancy between the increase of foxes with MLL reported by questionnaire and the relative decrease of submission of mangy foxes in the framework of general health surveillance (Figs. 2, 3, 4) is largely due to the end of the research project on mange in 2005 [4] and the consequent increased awareness for sarcoptic mange, which is relatively easy to recognize in the field. Field partners continued to report the occurrence of the disease in the questionnaire survey without sending foxes to the FIWI for post-mortem examination.

### Camera-trapping

**Fig. 5:**
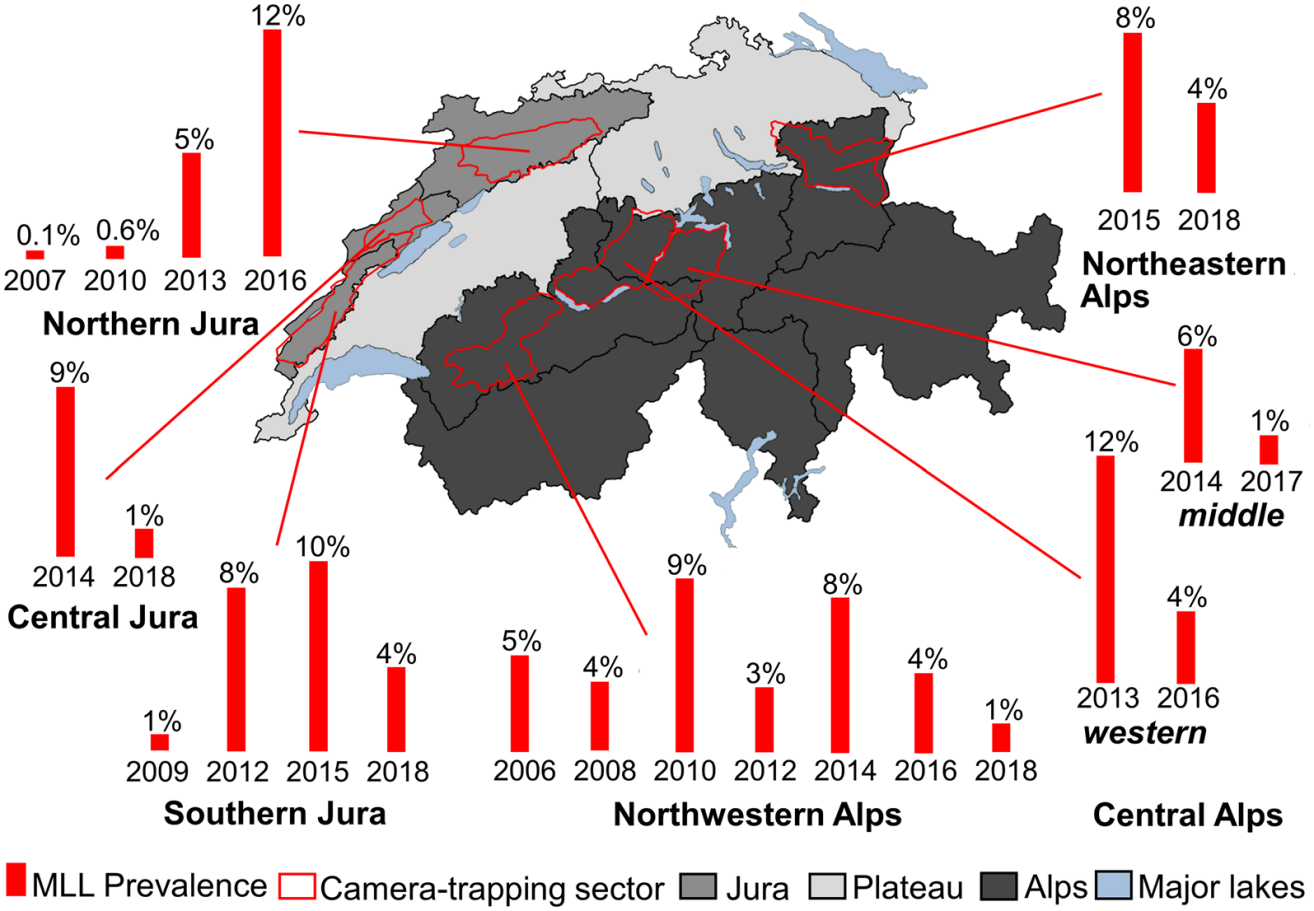
Prevalence of mange-like lesions in red foxes estimated from camera-trap data from 2005 to 2018. The pictures of red foxes (*Vulpes vulpes*) used were bycatch material from camera-trapping work performed by KORA from 2005 to 2018. The prevalence of mange-like lesions is indicated for each of the 23 trapping sessions (one session = 60 days in winter) in each of the 7 sectors considered for this study. Years indicated under the bars correspond to the year of the end of the session. Shades of grey refer to the three biogeographical regions. Biogeographical subregions are delimited by black lines. *Abbreviation*: MLL, mange-like lesions

MLL were recognizable in 6% of the fox pictures (Additional file 1: Table S2). In the Northwestern Alps sector, MLL prevalence showed a sinusoidal trend ranging from 1% (95% CI: 1–2%) to 9% (95% CI: 8–12%) between 2006 and 2018, suggesting an endemic situation (Fig. 5, Additional file 1: Table S2). The differences of MLL prevalence between pairs of successive sessions were almost all statistically significant (Additional file 1: Table S2). In the two Central Alps sectors, MLL prevalence decreased significantly from 6% (95% CI: 4–7%) and 12% (95% CI: 10–13%) in 2013–2014 to 1% (95% CI: 1–2%; TEP: *χ*^2^ = 36.086, *df* = 1, *P* < 0.0001) and 4% (95% CI: 3–5%; TEP: *χ*^2^ = 92.84, *df* = 1, *P* < 0.0001) in 2016–2017, respectively (Fig. 5, Additional file 1: Table S2). In the Northeastern Alps sector, MLL prevalence significantly decreased from 8% (95% CI: 7–9%) in 2015 to 4% in 2018 (95% CI: 3–6%; TEP: *χ*^2^ = 14.956, *df* = 1, *P* = 0.00011; Fig. 5, Additional file 1: Table S2).

In both the Northern and Southern Jura sectors, MLL prevalence remained very low (0.1–1%, 95% CI: 0–2%) from 2007 to 2010 (Fig. 5). From 2010 to 2016, MLL prevalence continuously increased in all three subregions of the Jura (Fig. 5, Additional file 1: Table S2), suggesting an epidemic. In the last session (2018), MLL prevalence dropped significantly in the Southern Jura (TEP: *χ*^2^ = 48.218, *df* = 1, *P* < 0.0001) and the Central Jura sectors (TEP: *χ*^2^ = 43.775, *df* = 1, *P* < 0.0001), suggesting a return to an endemic situation (Fig. 5, Additional file 1: Table S2).

### Hunting statistics

The national hunting bag increased from 1958 to 1967, i.e. before the epidemic wave of rabies reached Switzerland, decreased then during the rabies epidemics until 1984, exponentially re-increased until the 1990s, largely above the pre-rabies level and has been moderately re-decreasing since the mid-1990s (Fig. 2e, Additional file 2: Figure S2, Additional file 3: Figure S5, Additional file 5: Table S8). Similarly, the number of foxes found dead has continuously increased since 1968 (Fig. 2e), the year these data were recorded for the first time. During the pre-rabies phase, HIPDs were highest in the northeastern part of Switzerland and lowest in the central and southern parts of Switzerland (Additional file 5: Table S8). During the rabies epidemic, HIPDs decreased in cantons where rabies prevalence was higher and local rabies outbreaks lasted longer and where sarcoptic mange apparently disappeared (e.g. Jura, Plateau and Eastern Alps [50]; Additional file 5: Table S8). Following the rabies epidemics, HIPDs remained low in cantons where rabies did not arrive and sarcoptic mange did not disappear (part of the Alps) but had increased again in cantons where sarcoptic mange re-emerged and spread further (Jura and Plateau; Additional file 5: Table S8).

## Discussion

In this study, a multi-method approach was used to describe the spatiotemporal pattern of spread of sarcoptic mange in foxes in Switzerland since the 19th century. In relation to the massive sylvatic rabies epidemic that spread through Europe in the second half of the 20th century [62, 63], long-term dynamics of sarcoptic mange in Switzerland can be roughly subdivided into three phases: (i) pre-rabies (until the 1960s); (ii) rabies epidemic peak (1970s–1980s); and (iii) post-rabies peak (1990s–2010s), i.e. phase-out (until 1997) and post-eradication (from 1999 onwards). Sarcoptic mange has been present in free-ranging wildlife for decades and perhaps even centuries in Switzerland, in agreement with previous reports [11, 57, 64, 65]. The widespread distribution of sarcoptic mange in a few isolated districts or municipalities before the arrival of rabies is consistent with an endemic situation, as previously mentioned [57, 65–68]. Rabies entered the northeastern part of Switzerland in 1967 and subsequently spread further as a large closed front wave over almost the northmost half of the country, especially over the Jura and the Plateau (Fig. 3) [51]. Since then, awareness, surveillance efforts and research interest for sarcoptic mange increased resulting in an improved data accuracy and finer scale resolution. The disease became less common and apparently disappeared (“hypoendemic” situation) in subregions with rabies occurrence but largely persisted in the subregions without rabies occurrence (Southern Alps, Southwestern Alps, parts of the Central Alps). In the post-rabies peak phase, sarcoptic mange re-emerged where it had apparently disappeared and spread epidemically until occurring in almost all of the country. The epidemic front of sarcoptic mange seemed to move from the Alps towards the Jura following the “retreat line” of rabies. Since the mid-2010s, a mosaic of endemic and epidemic situations has been observed in the most recently affected subregions (rest of the Alps, Plateau and Jura). Therefore, in contrast to the perception in the late 1990s, sarcoptic mange did not newly emerge in Switzerland but, rather, re-emerged especially in the Northwestern and Central Alps.

The findings of this study suggest a series of epidemiological transitions: from an endemic to a “hypoendemic” and epidemic situation first and from an epidemic back to a partially endemic situation then. Patterns of disease spread are multifactorial and therefore difficult to be completely elucidated, especially in a highly adaptable species such as the red fox (e.g. [69]). Influencing factors may include variations in host population density, host population dynamics, social, behavioural and spatial ecology (including dispersal, migration and changes in home range sizes), as well as by various other factors including landscape structure, environmental and climatic factors, host(s) life history, and host and pathogen genetic diversity [11, 70–74]. It can be hypothesised that the described epidemiological transitions may have been eased by three successive processes.

First, rabies epidemic and related control interventions (e.g. increased hunting pressure and den gassing) led to a demographic bottleneck characterized by a fox population decline of up to 80–90% [43, 50, 75, 76] in parallel to a strong reduction or even apparent disappearance of sarcoptic mange. Demographic bottlenecks and population separations may lead to a decrease of genetic diversity [77]. This may limit the adaptive potential of populations to pathogens [78, 79] and, in turn, influence disease dynamics and increase vulnerability to diseases [80, 81]. The fox population collapse (already denominated as “rabies pit” [56]) in the Plateau and the Jura may have impaired, on the one hand, the transmission of *S. scabiei* due to the reduced density and therefore contacts among foxes and, on the other hand, removed diseased and also resistant individuals, disrupting the long-term host-parasite adaptation and generating a susceptible “naïve-like” population. At the same time, sarcoptic mange persisted endemically in most Alpine subregions, where local fox population may have acted as a reservoir for *S. scabiei*. In these subregions, HIPDs had been lowest since the 1930s [56] and pressure of rabies and related management measures were less important or not existing at all [51].

Secondly, sarcoptic mange re-emerged and spread with an epidemic wave during and after rabies eradication. The reason for this is likely multifactorial and difficult to analyse without a modelling approach. On the one hand, following the successful oral vaccination campaigns initiated in 1978, rabies was progressively eliminated [47, 50] and the fox population recovered as suggested by the large increase of the national hunting bag and roadkill numbers [43, 56]. The increased density of the mostly “naïve-like” fox population may have facilitated contacts among animals and therefore the transmission of *S. scabiei*, i.e. a local re-emergence of the disease. This increase of population density (as suggested by the HIPDs) has been most prominent in the Plateau and in the Jura, where rabies had persisted longest and therefore rabies-related mortality had been highest [50, 56] and where sarcoptic mange had apparently disappeared but finally re-emerged and further spread. The limited to absent increase of the HIPDs in the Southern, Central, Southeastern and Southwestern Alps [56], where sarcoptic mange has persisted endemically, may have been due to the low or absent rabies-related mortality and a lower carrying capacity than in the Jura and Plateau [56]. On the other hand, the occasional movement of infected animals to naïve-like subpopulations, e.g. through dispersal, could explain the observed epidemic wave. Since food availability and possibly the fox population density are lower in the Alps, home range sizes may be larger but overlap less than in the Jura and the Plateau [55, 69, 82]. Assuming negative density dependent dispersal in foxes [43, 83], more juvenile foxes would leave their natal range and those that disperse would move over longer distances in the affected Alpine subregions (lower density) than in the Plateau and the Jura (higher density). As a result, the prevalence of sarcoptic mange likely increased while the spread of the epidemic front decelerated in areas with higher fox densities such as the Plateau and the Jura. The observed pattern contrasts with the relatively rapid epidemic spread through Sweden, a country much larger than Switzerland, in the 1970s–1980s [84]. This difference is attributable to several factors. Compared to Sweden, food availability is likely higher in Switzerland, resulting in higher fox densities and smaller home ranges. Habitat fragmentation is also higher [51, 85] and in the 1980s foxes started to colonize human settlements including large Swiss cities [11, 86]. Although Alpine valleys represent separate epidemiologic compartments [63], occasional movements of mangy foxes over natural barriers may have occurred [51] and might have contributed to the spread of sarcoptic mange northwards [11]. Finally, climate change in Switzerland, characterized by the increase of yearly mean temperature, decrease of snow cover and fluctuations in the yearly precipitations since the 1980s [87], might have provided a suitable fox habitat at higher altitude, as it has been suspected in Italy [88], and influenced the ecology of *S. scabiei*.

Thirdly, while the epidemic spread was still ongoing in 2017–2018, especially in the Eastern Plateau, the disease may have already returned to an endemic status in subregions where it had re-emerged first, due to years of host-pathogen adaptation [89], i.e. through selection of more resistant individuals and some degree of immunity [21, 90]. This process may take a relatively long time to occur [11, 13, 90, 91], as reported for example in Scandinavia, where more than two decades were needed for sarcoptic mange to become endemic after its emergence and following epidemic spread [89].

Infectious diseases such as sarcoptic mange [7, 63, 92], canine distemper [93–95] and rabies [76] can drive wild canid population dynamics by altering population size, growth rate, animal distribution, migration patterns and genetic diversity [96–98]. Studies on interactions between diseases [99, 100] in wildlife are rare, but comparable interactions between distemper and sarcoptic mange were hypothesized in wolves (*Canis lupus*) in the USA [95] and in Spain [101], while an association between genetic structure of fox population and infection with distemper and rabies was observed in Northern Italy [70]. This study suggests that rabies-related changes in the fox population dynamics affected the dynamics of sarcoptic mange, a phenomenon that has also recently been observed in Estonia [102–104].

A multi-method and a long-term approach were used to successfully compensate the limitations and exploit the advantages of different data sources, to cross-validate their results and to detect trends of disease dynamics that would have been misinterpreted or not recognized if shorter study periods had been considered [105]. All five data sources converged towards similar trends, complementing and overlapping each other, both geographically and in time. Improvement of wildlife disease surveillance has progressively delivered better information, demonstrating the relevance of wildlife health surveillance programmes to produce reliable information on wildlife disease epidemiology, which allows informed management decisions [105, 106].

Although the strength of this study lies in the combination of multiple data sources, each single method had limitations. The main limitation of the review of the historical literature included the difficulty to detect all existing documents due to lacking databases [107]. The use of HIPD is controversial but can be useful to evaluate long-term population trends in large areas [108, 109]. Limitations of the general health surveillance programme were, as already known for wildlife populations, the discovery, selection and submission of carcasses by field partners operating in different cantons, personnel changes and varying levels of disease awareness [6, 46, 105]. Sarcoptic mange can only be confirmed through isolation and identification of *S. scabiei* [109]. This was possible only for dead foxes submitted in the framework of the general health surveillance programme at the FIWI and of the rabies eradication campaign at the SRC. The other methods could only rely on MLL as indicator of sarcoptic mange (syndromic approach), which implies a risk of diagnostic error. However, rather typical skin lesions and confirmation of mitesʼ presence in foxes submitted from affected locations strongly suggest that the observed MLL were (at least mostly) due to the spread of *S. scabiei* throughout the fox population. The data obtained in the context of the rabies eradication campaign provided the most representative and precise information about the spatiotemporal spread of sarcoptic mange but only during a relatively limited time-period (24 years) compared to the general health surveillance at the FIWI (60 years).

To our knowledge, this was the first long-term (almost 20 years) questionnaire survey on the occurrence of MLL worldwide. The advantage of repetitive assessments was the participation of experienced field partners, while main limitations were the size differences of the districts and the limited accuracy and representativeness of MLL reports before the first survey round (2001). Nevertheless, the strength of such surveys is that they are cheap, applicable to the whole country and easily repeatable. Although camera-trapping has already been used to detect MLL occurrence in wildlife [19, 110, 111], this is the first long-term assessment (15 years) of mange occurrence using bycatch camera-trap pictures collected during repetitive sessions in selected sectors. This constancy allowed estimating MLL prevalence and its temporal trend. The data were precise at local level and, although not extrapolable to the entire country, they demonstrated clear differences among sectors. Detectability of MLL depends on many factors including characteristics and height of camera traps, image quality and behavioural changes in mangy foxes [11, 112, 113]. However, we expect that these aspects did not influence the detected trends as all sessions in a given camera-trapping sector would have been equally affected. The high time investment, material and personnel costs can limit the use of camera-trapping solely for monitoring MLL occurrence and highlight the utility of bycatch data for this use.

## Conclusions

Sarcoptic mange in red foxes has likely been endemic in Switzerland as well as in other European countries at least since the mid-19th century. The rabies epidemics seem to have influenced the pattern of spread of mange in several locations, revealing an interesting example of disease interaction in free-ranging wildlife. This phenomenon may have been related to susceptibility changes in the population due to the disruption of the host-parasite co-evolution process within the fox population and to variation of animal numbers. The combination of multiple surveillance tools to study the long-term dynamics of sarcoptic mange in the red fox in Switzerland proved to be a successful investigation strategy. Considering the advantages and disadvantages of the applied methods, the cross-validation of the results indicate that a questionnaire survey is a particularly efficient and low-cost option to monitor the spatiotemporal spread of sarcoptic mange, assuming a uniform disease awareness and a good collaboration of field partners throughout the study area.

## Supplementary information

**Additional file 1: Table S1.** Features of the camera-trapping monitoring. **Table S2.** Overview of data obtained from 23 camera-trapping sessions.

**Additional file 2: Table S3.** Cantonal occurrence of sarcoptic mange in foxes examined in the framework of general health surveillance for wildlife at the FIWI (1958–2018). **Table S4.** Yearly occurrence of sarcoptic mange in foxes examined at the FIWI. **Figure S1.** Monthly distribution of foxes with mange-like lesions examined at the FIWI. **Figure S2.** Number of foxes found dead and culled in Switzerland and of foxes examined per year at the FIWI. **Figure S3.** Number of foxes examined per period at the FIWI. **Figure S4.** Spatiotemporal distribution of foxes with sarcoptic mange examined at the FIWI (1958–2017).

**Additional file 3: Table S5.** Spatiotemporal distribution of foxes with sarcoptic mange and rabies (1967–1990). **Figure S5.** Foxes with sarcoptic mange analysed per year at the SRC. **Figure S6.** Spatiotemporal distribution of foxes with sarcoptic mange and/or rabies (1967–1990). **Figure S7.** Sarcoptic mange and rabies spread in Switzerland (1967–1992).

**Additional file 4: Table S6.** Answers to the questionnaire survey on sarcoptic mange in foxes per year (<1980–2017). **Table S7**. Answers to the questionnaire survey on sarcoptic mange in foxes per Swiss canton (<1980–2017). **Figure S8.** Changes in the occurrence of foxes with mange-like lesions in districts of surveillance per surface area. **Figure S9.** Temporal occurrence of mange-like lesions in foxes per number of cantons and districts of surveillance (questionnaire survey <1980–2017). **Figure S10.** Reliability categories of sarcoptic mange diagnosis in foxes (questionnaire survey 2001–2017). **Figure S11.** Trends in the occurrence of mange-like lesions in foxes in Switzerland (questionnaire survey 2001–2017).

**Additional file 5: Table S8**. Cantonal and national hunting statistics before, during and after the rabies epidemics.

## Data Availability

All relevant data are presented in the article. The original datasets are available from the corresponding author upon reasonable request.

## Abbreviations

CI: confidence interval
CSA: country surface area
FIWI: Centre for Fish and Wildlife Health
HIPD: hunting indicator of population density
MLL: mange-like lesions
SRC: Swiss Rabies Centre
TEP: test of equal proportions
