# Supplementary material for: Spatiotemporal spread of sarcoptic mange in the red fox (Vulpes vulpes) in Switzerland over more than 60 years: lessons learnt from comparative analysis of multiple surveillance tools
Source: Parasit Vectors. 2019 Nov 5;12:521. doi: 10.1186/s13071-019-3762-7 (PMC6833187; doi:10.1186/s13071-019-3762-7)
Supplement: Supplementary file 1 — Additional file 1: Table S1. Features of the camera-trapping monitoring. Table S2. Overview of data obtained from 23 camera-trapping sessions. [file 13071_2019_3762_MOESM1_ESM.pdf]

Additional file 1

Camera-trapping

Source: database of the KORA – Carnivore Ecology and Wildlife Management

Table S1. Features of the camera-trapping monitoring

Originally designed by the KORA for monitoring the Eurasian lynx (*Lynx lynx*) population in Switzerland, this camera trapping work also produced bycatch data, which were used to estimate the prevalence of mange-like lesions in red foxes (*Vulpes vulpes*) in seven sectors. The table includes the location of the study sector (biogeographical subregion), its surface area (km²), the time interval between sessions (years), the number of sessions performed, the period including all sessions performed in the given sector, the months during which sessions were performed (phase 1: December–February; phase 2: February–April), the type of camera-trap used (analog, digital), the number of camera-trap sites, the number of different collaborators who assessed the pictures, the total number of pictures obtained and the number of pictures of foxes. *Abbreviations*: a, analog; Collab, collaborators; d, digital; pic, pictures.

Biogeographical subregion	Area (km²)	Interval (y)	Session (n)	Period (y)	Phase	Type	Sites (n)	Collab (n)	All pic (n)	Fox pic (n)
1.1 Southern Jura	728	3	4	2008-18	1, 2	a, d	49	17	13,435	6212
1.2 Central Jura	236	4	2	2014-18	1, 2	d	16	6	3090	1372
1.3 Northern Jura	882	3	4	2007-16	1, 2	a, d	50	7	11,034	3964
3.1 Northwestern Alps	861	2	7	2005-18	1, 2	a, d	46	14	19,254	9225
3.2.1 Central Alps – West	705	3	2	2013-16	1, 2	d	74	6	10,991	4211
3.2.2 Central Alps – Middle	951	3	2	2013-17	1	d	38	9	4710	2428
3.3 Northeastern Alps	871	3	2	2014-18	1	d	54	10	6602	2756
Total	5234	-	23	2005-18	-	-	327	33	69,116	30,168

Table S2. Overview of data obtained from 23 camera-trapping sessions

The table indicate the total number of pictures collected in 7 sectors from 2005 to 2018 (source: database of KORA), the number and percentage of pictures of red foxes (*Vulpes vulpes*) obtained, the apparent prevalence (with 95% confidence interval) of mange-like lesions among these foxes, and the number and percentage of sites where pictures of foxes with mange-like lesions were taken. *P* values were calculated with the test of equal proportion for comparing the prevalence and the percentage of sites with mange-like lesions foxes, respectively, between pairs of consecutive sessions. The year used to designate a session corresponded to the year when the session ended. *Abbreviations*: CI, confidence interval; MLL, mange-like lesions; *P*, *P* value: nsd, no statistical difference, $P > 0.05$; *, $P < 0.05$; **, $P < 0.01$; ***, $P < 0.001$; ****, $P < 0.0001$.

Sector / Session	Pictures Total (n)	Fox (n,%)	MLL (n,%)	95% CI	*P*	Sites Fox (n,%)	MLL (n,%)	*P*
1.1 Southern Jura								
2009	1248	647 (52%)	6 (1%)	0.3-2%	/	42 (88%)	3 (7%)	/
2012	4715	1846 (39%)	155 (8%)	7-10%	****	49 (100%)	22 (45%)	***
2015	3846	1909 (50%)	184 (10%)	8-11%	nsd	48 (98%)	20 (42%)	nsd
2018	3965	1938 (49%)	87 (5%)	36-55%	****	48 (100%)	16 (33%)	nsd
1.2 Central Jura								
2014	1398	609 (44%)	52 (9%)	6-11%	/	16 (100%)	8 (50%)	/
2018	1692	791 (47%)	10 (1%)	0.6-2%	****	13 (81%)	1 (8%)	*
1.3 Northern Jura								
2007	2772	669 (24%)	1 (0.2%)	0.0-1%	/	43 (88%)	1 (2%)	/
2010	3063	1040 (34%)	6 (0.6%)	0.2-1%	nsd	48 (98%)	5 (10%)	nsd
2013	2366	1424 (60%)	74 (5%)	4-7%	****	50 (100%)	20 (40%)	***
2016	2833	831 (29%)	96 (12%)	10-14%	****	49 (98%)	14 (29%)	nsd
3.1 Northwestern Alps								
2006	1703	891 (52%)	43 (5%)	4-6%	/	46 (100%)	21 (46%)	/
2008	1844	966 (52%)	34 (4%)	2-5%	nsd	44 (96%)	7 (16%)	****
2010	2428	815 (34%)	71 (9%)	8-12%	****	41 (98%)	12 (29%)	nsd
2012	3098	1626 (53%)	54 (3%)	3-4%	****	44 (96%)	12 (27%)	nsd
2014	3418	1859 (54%)	147 (8%)	7-9%	****	46 (100%)	4 (9%)	*
2016	2694	1432 (53%)	57 (4%)	3-5%	****	45 (98%)	8 (18%)	nsd
2018	4069	1636 (40%)	19 (1%)	0.7-2%	****	46 (100%)	6 (13%)	nsd
3.2.1 Central Alps – Middle sector								
2014	1628	789 (49%)	44 (6%)	4-7%	/	36 (95%)	3 (8%)	/
2017	3082	1639 (53%)	21 (1%)	0.8-2%	****	38 (100%)	2 (5%)	nsd
3.2.2 Central Alps – Western sector								
2013	5608	2101 (38%)	243 (12%)	10-13%	/	74 (100%)	30 (41%)	/
2016	5383	2110 (39%)	77 (4%)	3-5%	****	73 (99%)	16 (22%)	*
3.3 Northeastern Alps								
2015	3408	1400 (41%)	113 (8%)	7-9%	/	54 (100%)	8 (15%)	/
2018	3194	1356 (43%)	60 (4%)	3-6%	***	54 (100%)	7 (13%)	nsd
Total								
2006-2018	69,116	27,740 (40%)	1578 (6%)	/	/	/	/	/
