# Supplementary material for: Spatiotemporal spread of sarcoptic mange in the red fox (Vulpes vulpes) in Switzerland over more than 60 years: lessons learnt from comparative analysis of multiple surveillance tools
Source: Parasit Vectors. 2019 Nov 5;12:521. doi: 10.1186/s13071-019-3762-7 (PMC6833187; doi:10.1186/s13071-019-3762-7)

Additional file 2

General Health Surveillance

Source: database of the Centre for Fish and Wildlife Health (FIWI)

Table S3. Cantonal occurrence of sarcoptic mange in foxes examined in the framework of the general health surveillance for wildlife at the FIWI (1958-2018)

Number of red foxes (*Vulpes vulpes*) examined, number and percentage of foxes with mange-like lesions and confirmed sarcoptic mange (*S. scabiei* infestation). *Abbreviation*: MLL, mange-like lesions.

Canton	Necropsy (n)	MLL (n)	*S. scabiei* (n)	*S. scabiei* (%)
Aargau	62	6	4	6
Appenzell Innerhoden	2	2	2	100
Appenzell Ausserrhoden	0	0	0	0
Bern	300	115	96	32
Basel-Landschaft	22	7	7	32
Basel-Stadt	62	12	9	15
Fribourg	131	43	40	31
Geneva	40	8	7	18
Glarus	8	2	2	25
Graubünden	132	22	19	14
Jura	19	2	1	5
Luzern	40	6	5	13
Neuchâtel	7	2	2	29
Nidwalden	4	2	2	50
Obwalden	12	6	4	33
Sankt Gallen	58	10	9	16
Schaffhausen	19	2	2	11
Solothurn	34	7	6	18
Schwyz	14	0	0	0
Thurgau	7	2	1	14
Ticino	13	8	8	62
Uri	13	4	3	23
Vaud	18	4	4	22
Wallis	90	51	43	48
Zug	7	0	0	0
Zürich	11	3	2	18

Table S4. Yearly occurrence of sarcoptic mange in foxes examined at the FIWI

Number, sex, age and number of cantons of origin of all red foxes (*Vulpes vulpes*) examined, of foxes with mange-like lesions, and of foxes with confirmed sarcoptic mange (*S. scabiei* infestation). *Abbreviations*: A, adult; C, cantons; F, female; J, juvenile; MLL, mange-like lesions; M, male; NA: data not available.

	Foxes (n)								MLL (n)								*S. scabiei* (n)							
	Total	F	M	NA	A	J	NA	C	Total	F	M	NA	A	J	NA	C	Total	F	M	NA	A	J	NA	C
2018	45	16	27	2	43	1	1	8	6	2	3	1	5	1	0	4	6	2	3	1	5	1	0	4
2017	14	8	4	2	9	3	2	8	0	0	0	0	0	0	0	0	0	0	0	0	0	0	0	0
2016	16	7	9	0	12	4	0	7	0	0	0	0	0	0	0	0	0	0	0	0	0	0	0	0
2015	41	15	26	0	32	9	0	11	4	2	2	0	4	0	0	4	3	1	2	0	3	0	0	3
2014	31	18	11	2	28	3	0	14	5	2	3	0	5	0	0	5	4	1	3	0	4	0	0	4
2013	35	11	19	5	33	2	0	12	7	1	5	1	7	0	0	4	6	1	4	1	6	0	0	4
2012	40	19	16	5	30	9	1	12	10	4	5	1	9	1	0	5	9	3	5	1	8	1	0	5
2011	50	24	26	0	39	10	1	13	7	2	5	0	7	0	0	3	6	1	5	0	6	0	0	3
2010	105	53	49	3	91	14	0	14	24	14	10	0	23	1	0	11	22	13	9	0	21	1	0	10
2009	65	31	32	2	47	17	1	15	22	12	9	1	19	3	0	9	19	10	8	1	16	3	0	9
2008	25	12	12	1	18	5	2	12	16	5	10	1	12	2	2	8	14	4	9	1	10	2	2	8
2007	26	15	11	0	23	3	0	12	7	4	3	0	7	0	0	4	7	4	3	0	7	0	0	4
2006	31	16	15	0	30	1	0	9	14	8	6	0	14	0	0	4	12	8	4	0	12	0	0	4
2005	223	97	124	2	193	30	0	12	129	57	71	1	124	5	0	7	110	47	62	1	105	5	0	7
2004	47	22	23	2	45	2	0	7	21	11	8	2	20	1	0	5	17	10	7	0	16	1	0	4
2003	7	5	2	0	4	3	0	5	2	2	0	0	1	1	0	2	1	1	0	0	0	1	0	1
2002	18	9	7	2	16	1	1	5	1	0	1	0	1	0	0	1	1	0	1	0	1	0	0	1
2001	16	8	8	0	13	0	3	8	1	1	0	0	1	0	0	1	1	1	0	0	1	0	0	1
2000	10	4	6	0	9	1	0	9	3	1	2	0	2	1	0	3	3	1	2	0	2	1	0	3
1999	14	6	7	1	11	2	1	8	3	0	2	1	3	0	0	2	2	0	1	1	2	0	0	2
1998	27	13	14	0	25	1	1	9	2	1	1	0	2	0	0	1	1	1	0	0	1	0	0	1
1997	8	3	5	0	8	0	0	1	2	1	1	0	2	0	0	1	1	0	1	0	1	0	0	1
1996	11	2	9	0	8	3	0	6	2	0	2	0	2	0	0	2	2	0	2	0	2	0	0	2
1995	6	4	2	0	4	2	0	5	1	1	0	0	1	0	0	1	1	1	0	0	1	0	0	1
1994	9	7	2	0	7	2	0	7	1	1	0	0	1	0	0	1	1	1	0	0	1	0	0	1
1993	11	4	7	0	10	1	0	7	1	0	1	0	1	0	0	1	0	0	0	0	0	0	0	0
1992	2	0	2	0	2	0	0	2	1	0	1	0	1	0	0	1	1	0	1	0	1	0	0	1
1991	12	4	7	1	10	0	2	5	0	0	0	0	0	0	0	0	0	0	0	0	0	0	0	0
1990	11	6	2	3	6	1	4	5	1	1	0	0	1	0	0	1	1	1	0	0	1	0	0	1
1989	11	4	7	0	7	4	0	5	0	0	0	0	0	0	0	0	0	0	0	0	0	0	0	0
1988	5	1	3	1	4	1	0	2	0	0	0	0	0	0	0	0	0	0	0	0	0	0	0	0
1987	4	1	2	1	3	1	0	1	0	0	0	0	0	0	0	0	0	0	0	0	0	0	0	0
1986	1	0	0	1	0	1	0	1	0	0	0	0	0	0	0	0	0	0	0	0	0	0	0	0
1985	2	1	1	0	1	1	0	2	0	0	0	0	0	0	0	0	0	0	0	0	0	0	0	0
1984	3	2	0	1	2	0	1	3	0	0	0	0	0	0	0	0	0	0	0	0	0	0	0	0
1983	9	1	5	3	7	0	2	5	0	0	0	0	0	0	0	0	0	0	0	0	0	0	0	0
1982	NA	NA	NA	NA	NA	NA	NA	NA	NA	NA	NA	NA	NA	NA	NA	NA	NA	NA	NA	NA	NA	NA	NA	NA
1981	0	0	0	0	0	0	0	0	0	0	0	0	0	0	0	0	0	0	0	0	0	0	0	0
1980	1	1	0	0	0	1	0	1	0	0	0	0	0	0	0	0	0	0	0	0	0	0	0	0
1979	0	0	0	0	0	0	0	0	0	0	0	0	0	0	0	0	0	0	0	0	0	0	0	0
1978	2	0	2	0	2	0	0	1	0	0	0	0	0	0	0	0	0	0	0	0	0	0	0	0
1977	5	1	2	2	4	0	1	2	1	0	1	0	1	0	0	1	1	0	1	0	1	0	0	1
1976	12	1	10	1	8	0	4	2	3	0	3	0	3	0	0	1	3	0	3	0	3	0	0	1
1975	11	4	3	4	9	0	2	2	4	2	1	1	3	0	1	2	2	2	0	0	2	0	0	2
1974	11	2	5	4	7	0	4	3	4	1	1	2	3	0	1	2	4	1	1	2	3	0	1	2
1973	7	3	3	1	6	0	1	2	0	0	0	0	0	0	0	0	0	0	0	0	0	0	0	0
1972	11	4	7	0	10	0	1	1	1	1	0	0	1	0	0	1	0	0	0	0	0	0	0	0
1971	28	16	9	3	28	0	0	5	3	1	2	0	3	0	0	2	2	1	1	0	2	0	0	2
1970	2	1	0	1	2	0	0	1	0	0	0	0	0	0	0	0	0	0	0	0	0	0	0	0
1969	1	0	0	1	1	0	0	1	0	0	0	0	0	0	0	0	0	0	0	0	0	0	0	0
1968	8	6	1	1	4	0	4	2	1	1	0	0	1	0	0	1	1	1	0	0	1	0	0	1
1967	11	5	4	2	8	0	3	4	5	2	3	0	5	0	0	2	4	1	3	0	4	0	0	1
1966	13	7	6	0	7	0	6	3	4	3	1	0	2	0	2	3	4	3	1	0	2	0	2	3
1965	5	3	1	1	1	1	3	3	0	0	0	0	0	0	0	0	0	0	0	0	0	0	0	0
1964	1	1	0	0	1	0	0	1	1	1	0	0	1	0	0	1	1	1	0	0	1	0	0	1
1963	2	0	1	1	1	0	1	1	1	0	1	0	1	0	0	1	1	0	1	0	1	0	0	1
1962	0	0	0	0	0	0	0	0	0	0	0	0	0	0	0	0	0	0	0	0	0	0	0	0
1961	1	1	0	0	0	0	1	1	1	1	0	0	0	0	1	1	0	0	0	0	0	0	0	0
1960	2	0	1	1	2	0	0	1	1	0	1	0	1	0	0	1	1	0	1	0	1	0	0	1
1959	3	0	1	2	2	0	1	1	3	0	1	2	2	0	1	1	3	0	1	2	2	0	1	1
1958	0	0	0	0	0	0	0	0	0	0	0	0	0	0	0	0	0	0	0	0	0	0	0	0

Figure S1. Monthly distribution of foxes with mange-like lesions examined at the FIWI

Number and percentage of red foxes (*Vulpes vulpes*) with and without mange-like lesions analysed in the framework of the national general health surveillance programme for wildlife in Switzerland from 1958 to 2018. *Abbreviation*: MLL, mange-like lesions.

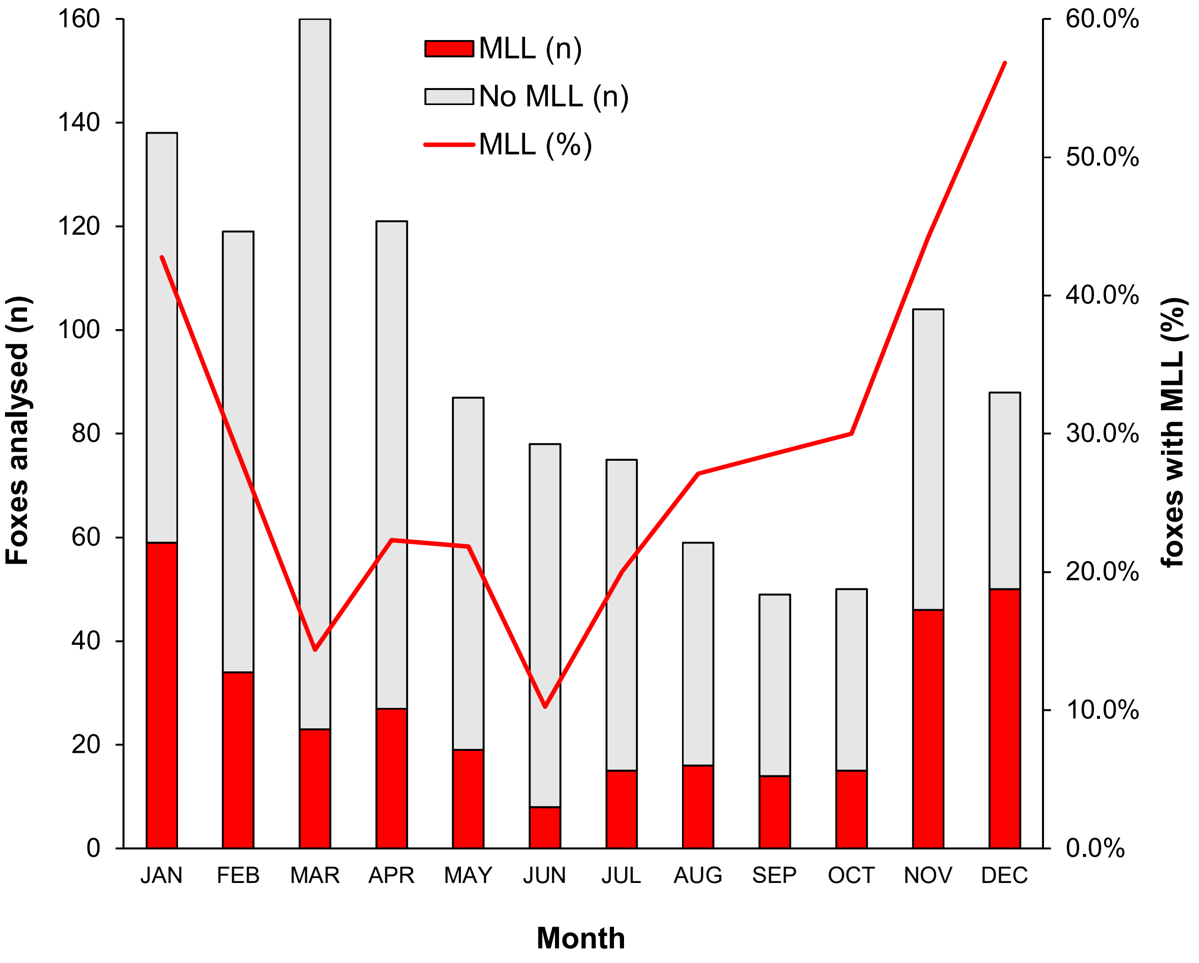

Figure S2. Number of foxes found dead and culled in Switzerland and number of foxes examined per year at the FIWI

a Number of red foxes (*Vulpes vulpes*) culled or found dead from 1958 to 2017 (source: national hunting statistics). **b** Number of foxes with or without mange-like lesions necropsied in the framework of the national general health surveillance programme for wildlife (source: database of the FIWI), the number of foxes tested positive for rabies at the Swiss Rabies Centre (Zanoni et al. 2000), and the number of foxes found dead with suspicion of disease (source: national hunting statistics; "Found dead with disease" refers to a category of the hunting statistics). The peak of necropsies in 2005 was due to a former research project on sarcoptic mange (Nimmervoll et al. 2013). The periods of rabies occurrence and of the national epidemics of canine distemper, both of which influenced the number of foxes submitted in the framework of the national wildlife health surveillance programme, are additionally indicated. *Abbreviation*: MLL, mange-like lesions.

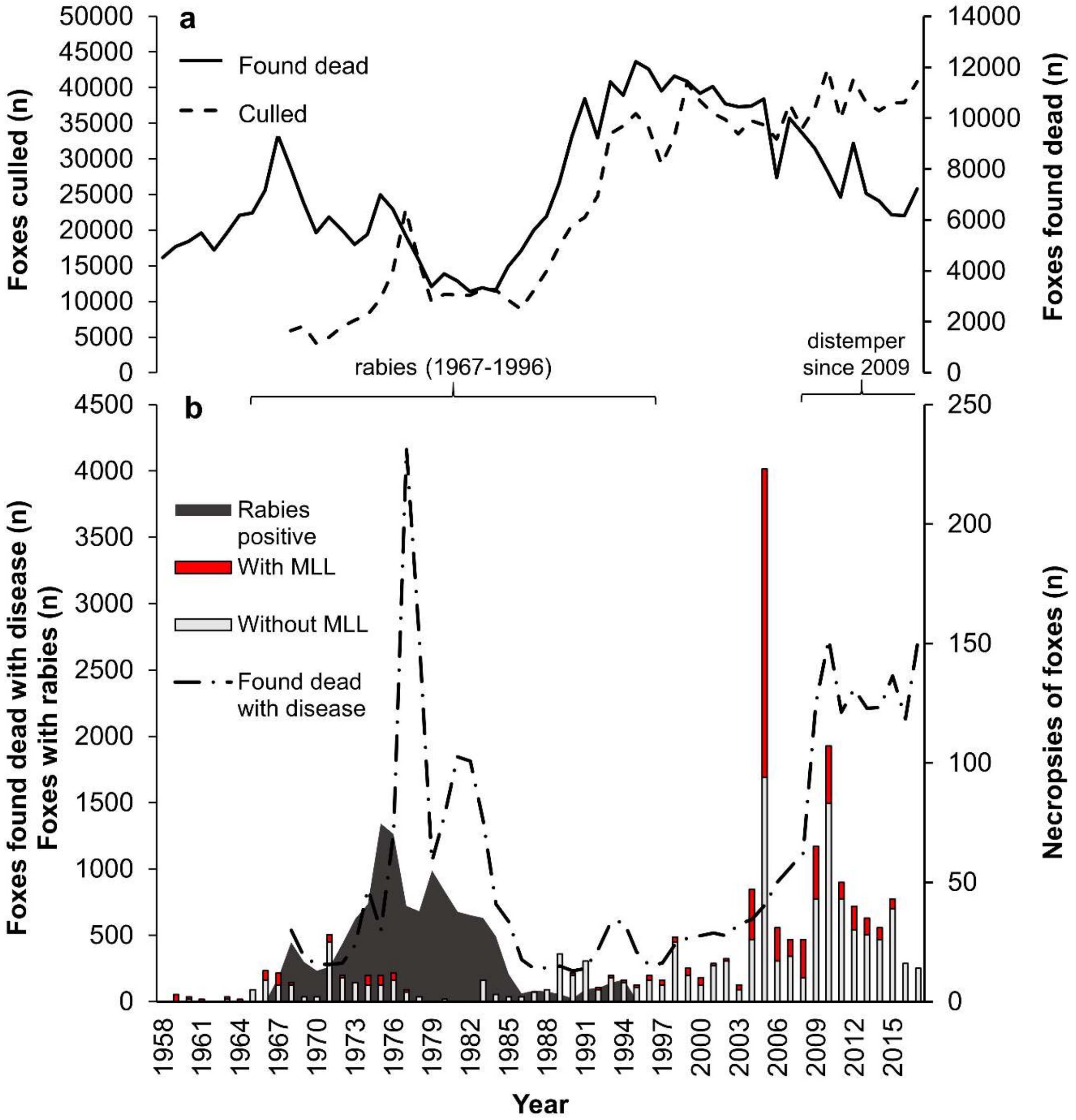

Figure S3. Number of foxes examined per period at the FIWI

Number of necropsies (*n* =1128) of red foxes (*Vulpes vulpes*) with (*n* =326) and without mange-like lesions carried out in Switzerland from 1958 to 2018. In foxes with mange-like lesions, sarcoptic mange was confirmed or not through the tentative detection of intradermal mites consistent with *Sarcoptes scabiei*. *Abbreviation*: MLL, mange-like lesions.

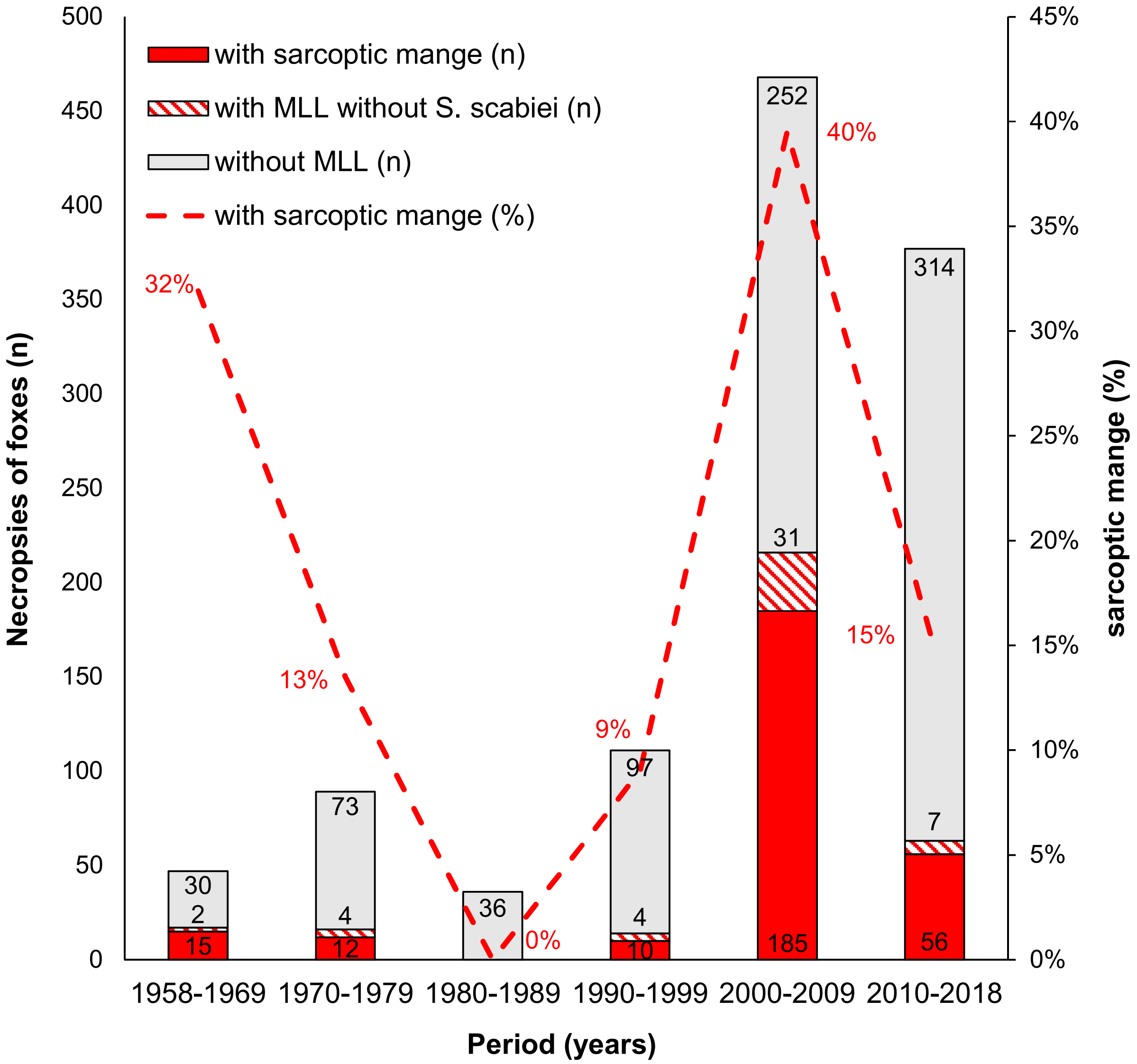

Figure S4. Spatiotemporal distribution of foxes with sarcoptic mange examined at the FIWI (1958-2017)

Districts of surveillance from where red foxes (*Vulpes vulpes*) were submitted in the framework of the national general health surveillance programme for wildlife are illustrated with different colors depending on whether foxes had sarcoptic mange or not. Sarcoptic mange was defined as the presence of mange-like lesions confirmed by the detection of *Sarcoptes scabiei*. The total number of foxes necropsied is indicated under the corresponding period. Pie charts represent the percentage of necropsied foxes with and without sarcoptic mange. Biogeographical subregions are delimited by black lines.

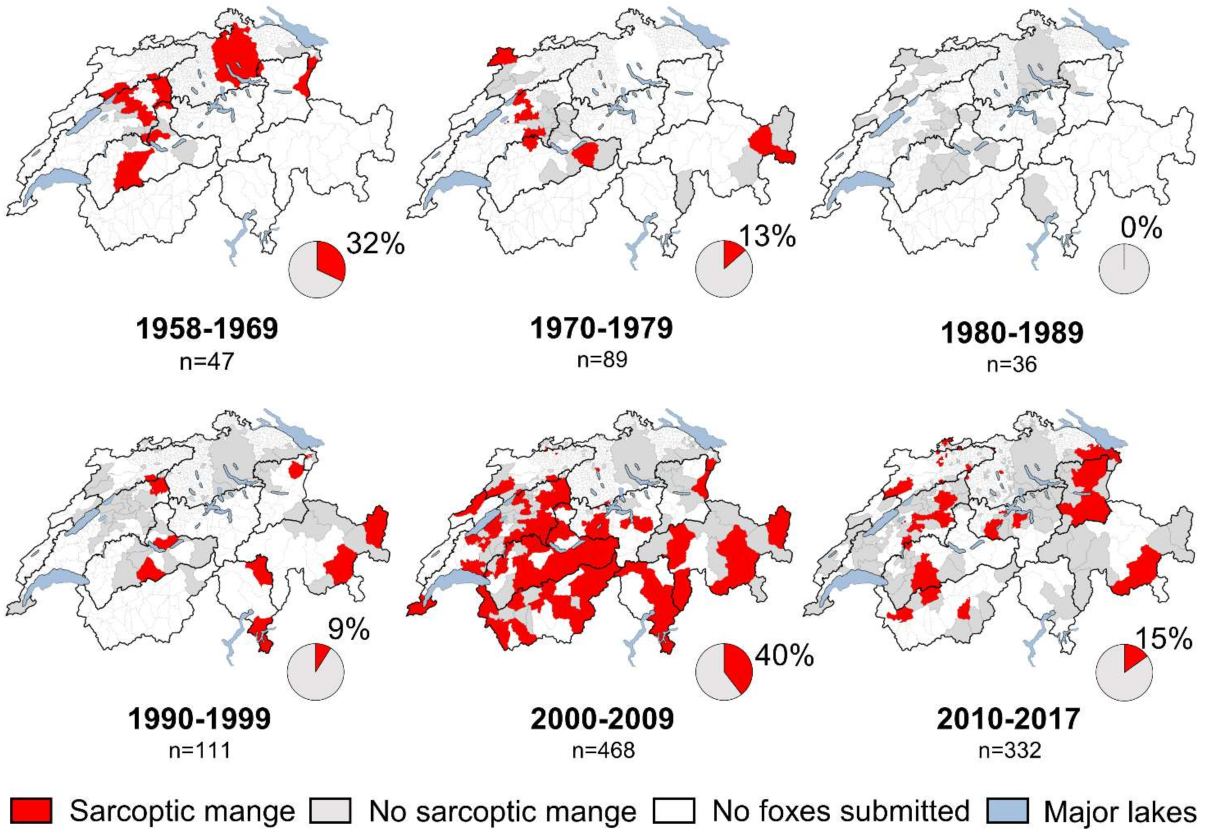

Supplement: Supplementary file 2 — Additional file 2: Table S3. Cantonal occurrence of sarcoptic mange in foxes examined in the framework of general health surveillance for wildlife at the FIWI (1958–2018). Table S4. Yearly occurrence of sarcoptic mange in foxes examined at the FIWI. Figure S1. Monthly distribution of foxes with mange-like lesions examined at the FIWI. Figure S2. Number of foxes found dead and culled in Switzerland and of foxes examined per year at the FIWI. Figure S3. Number of foxes examined per period at the FIWI. Figure S4. Spatiotemporal distribution of foxes with sarcoptic mange examined at the FIWI (1958–2017). [file 13071_2019_3762_MOESM2_ESM.pdf]
