# Supplementary material for: Spatiotemporal spread of sarcoptic mange in the red fox (Vulpes vulpes) in Switzerland over more than 60 years: lessons learnt from comparative analysis of multiple surveillance tools
Source: Parasit Vectors. 2019 Nov 5;12:521. doi: 10.1186/s13071-019-3762-7 (PMC6833187; doi:10.1186/s13071-019-3762-7)

Additional file 3

Data on mange collected during the campaign for sylvatic rabies eradication

Source: database of the Swiss Rabies Centre (SRC)

Table S5. Spatiotemporal distribution of foxes with sarcoptic mange and rabies (1967-1990)

Number, percentage, and geographical origin of red foxes (*Vulpes vulpes*) with sarcoptic mange and with rabies analysed at the Swiss Rabies Centre (SRC) in the framework of the rabies eradication campaign during five periods (1967, 1968-1974, 1975-1979, 1980-1984, 1985-1990). *Abbreviations*: nd, no data; SM, sarcoptic mange.

**Biogeographical Subregions**		**Total (1967-1990)**			**1967**		**1968-1974**		**1975-1979**		**1980-1984**		**1985-1990**	
		**Origin SM cases (%)**	**Foxes with SM**	**Foxes with Rabies**	**Foxes with SM**	**Foxes with Rabies**	**Foxes with SM**	**Foxes with Rabies**	**Foxes with SM**	**Foxes with Rabies**	**Foxes with SM**	**Foxes with Rabies**	**Foxes with SM**	**Foxes with Rabies**
1.	Jura	7%	20 (0.4%)	45.4%	nd	0.0%	13 (4.0%)	0.0%	5 (0.3%)	71.6%	0 (0.0%)	76.6%	2 (0.1%)	13.7%
1.1	Southern Jura	0%	0 (0.0%)	32.6%	nd	nd	nd	nd	0 (0.0%)	68.0%	0 (0.0%)	52.9%	0 (0.0%)	19.4%
1.2	Central Jura	0%	1 (0.2%)	44.1%	nd	nd	1 (12.5%)	0.0%	0 (0.0%)	67.7%	0 (0.0%)	74.6%	0 (0.0%)	18.2%
1.3	Northern Jura	7%	19 (0.5%)	45.0%	nd	0.0%	12 (3.8%)	0.0%	5 (0.3%)	72.4%	0 (0.0%)	77.3%	0 (0.1%)	12.3%
2	Plateau	23%	64 (0.4%)	36.9%	nd	31.5%	38 (1.0%)	41.9%	11 (0.3%)	60.8%	10 (0.4%)	52.4%	5 (0.1%)	4.5%
2.1	Western Plateau	7%	21 (0.6%)	30.5%	nd	0.0%	3 (6.8%)	0.0%	8 (0.8%)	44.4%	10 (1.1%)	53.8%	0 (0.0%)	6.2%
2.2	Eastern Plateau	15%	43 (0.4%)	39.4%	nd	32.3%	35 (1.0%)	42.4%	3 (0.1%)	66.5%	0 (0.0%)	48.3%	5 (0.2%)	3.7%
3	Alps	70%	199 (1.0%)	20.7%	nd	0.0%	31 (0.6%)	28.6%	28 (0.5%)	25.7%	46 (1.0%)	24.8%	94 (2.3%)	1.0%
3.1	Nortwestern Alps	3%	9 (0.7%)	33.5%	nd	0.0%	0 (0.0%)	0.0%	3 (0.4%)	56.2%	5 (0.7%)	34.3%	1 (0.4%)	0.0%
3.2	Central Alps	9%	25 (0.8%)	19.5%	nd	0.0%	13 (0.7%)	15.1%	5 (0.8%)	22.6%	4 (0.7%)	34.8%	3 (1.9%)	0.0%
3.3	Northeastern Alps	6%	16 (0.2%)	17.7%	nd	0.0%	4 (0.3%)	56.1%	0 (0.0%)	16.2%	0 (0.0%)	20.3%	12 (0.5%)	0.1%
3.4	Southwestern Alps	13%	37 (3.6%)	23.3%	nd	0.0%	0 (0.0%)	0.0%	13 (2.8%)	39.4%	12 (4.6%)	8.0%	12 (3.9%)	12.5%
3.5	Southern Alps	31%	88 (28.7%)	0.0%	nd	nd	nd	nd	0 (0.0%)	0.0%	24 (15.9%)	0.0%	64 (42.3%)	0.0%
3.6	Southeastern Alps	9%	24 (0.4%)	22.7%	nd	nd	nd	nd	nd	nd	1 (0.1%)	27.3%	2 (0.3%)	0.1%
Switzerland		100%	283 (0.7%)	30.2%	nd	27.3%	82 (0.9%)	33.0%	44 (0.4%)	46%	56 (0.7%)	38.9%	101 (1.0%)	4.8%

Figure S5. Foxes with sarcoptic mange analysed per year at the SRC

a Number of red foxes (*Vulpes vulpes*) culled and found dead from 1967 to 1990 in Switzerland (source: national hunting statistics). **b** Percentage of foxes with sarcoptic mange and the yearly number of foxes analysed at the Swiss Rabies Centre (SRC) during the same period (source: database of the SRC). Data on the presence of sarcoptic mange were not available for 1967, 1974 and 1977. *Abbreviation*: SM, sarcoptic mange.

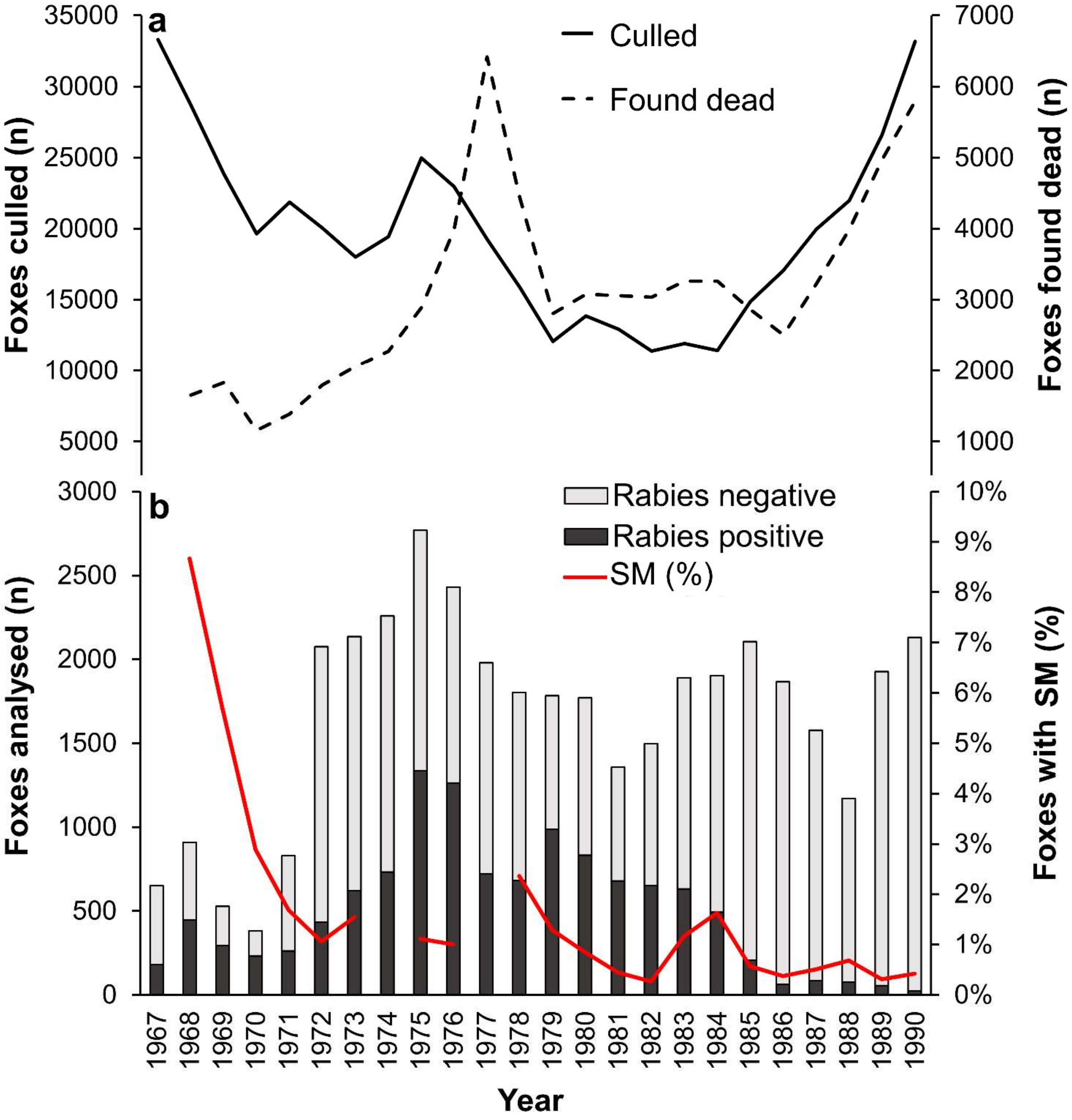

Figure S6. Spatiotemporal distribution of foxes with sarcoptic mange and/or rabies (1967-1990)

Municipalities from where red foxes (*Vulpes vulpes*) were submitted to the Swiss Rabies Centre (SRC) are illustrated with different colors depending on whether foxes had sarcoptic mange (*n* =283) or not and whether they were tested positive or negative for rabies (source: database of the SRC). Data on mange occurrence were not available for 1967, 1974 and 1977. The number of foxes with sarcoptic mange is indicated below the corresponding period. Pie charts indicate the percentages of the surface area of municipalities from where foxes with and without sarcoptic mange (rabies-negative or rabies-positive) were submitted. Biogeographical subregions are delimited by black lines. *Abbreviation*: SM, sarcoptic mange; pos, positive; neg, negative.

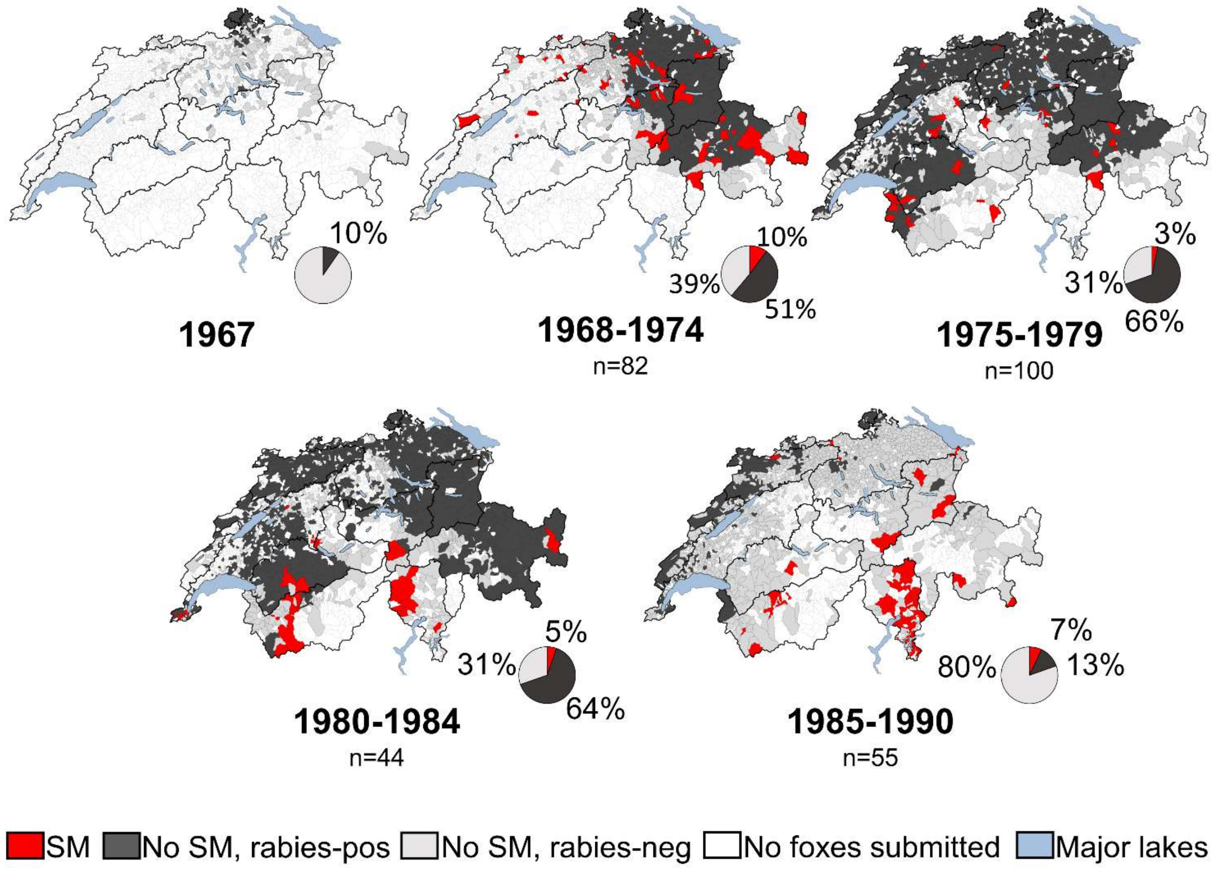

Figure S7. Sarcoptic mange and rabies spread in Switzerland (1967-1992)

This figure shows that the percentage of red foxes (*Vulpes vulpes*) with sarcoptic mange submitted to the Swiss Rabies Centre decreased as rabies spread across Switzerland. It also illustrates the difference in percentage of sarcoptic mange between the Southern Alps (data available since 1983) and the rest of the country. The spread of rabies is illustrated as the cumulative percentage of surface area with confirmed rabies occurrence. Only surface areas below 2000 meters above sea level were considered, in order to avoid an underestimation of the percentage of the surface area with rabies occurrence since fox habitat is typically located below that altitude and above it population density is too low for rabies persistence (Müller et al. 2000). *Abbreviation*: SM, sarcoptic mange.

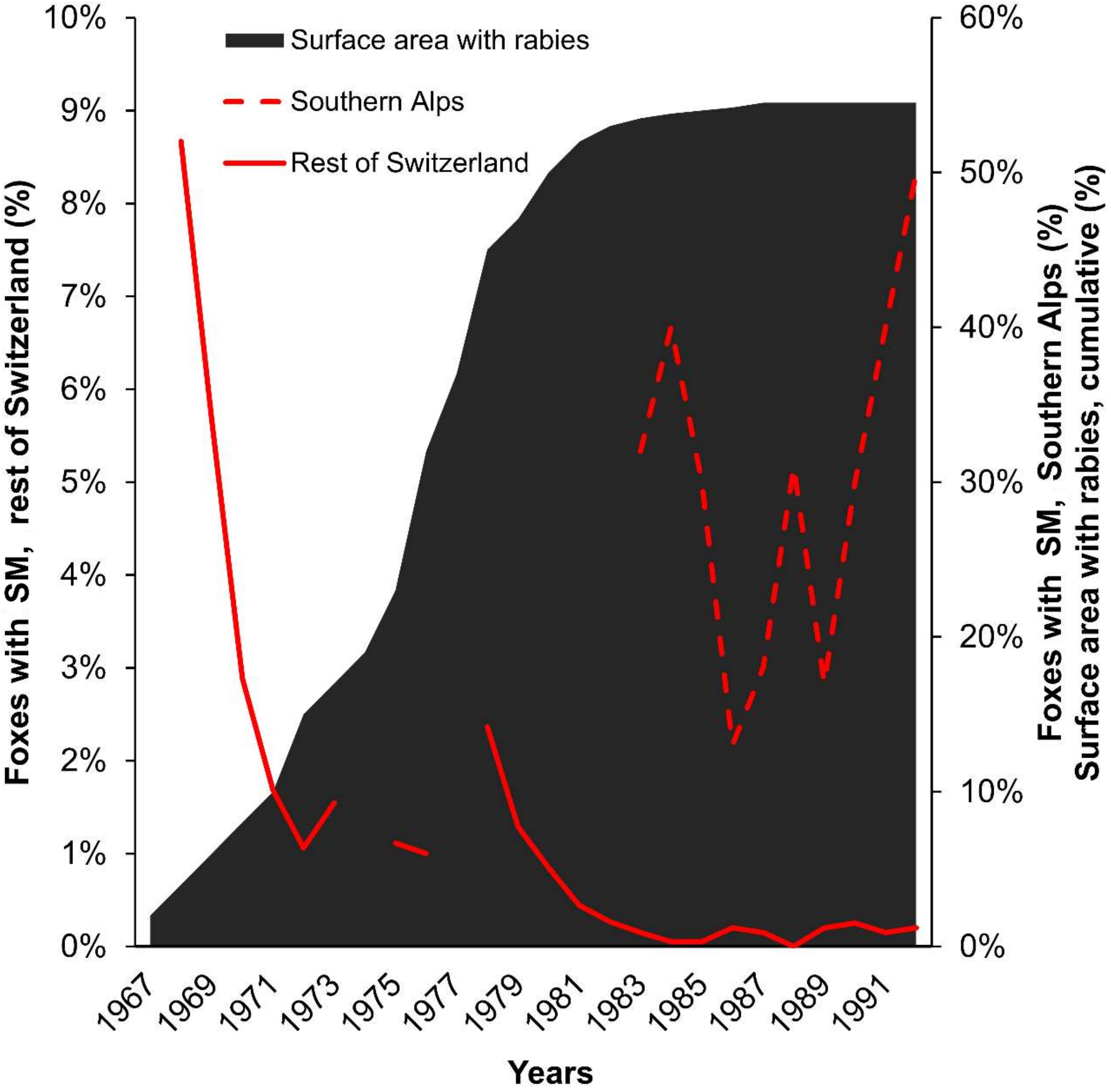

Supplement: Supplementary file 3 — Additional file 3: Table S5. Spatiotemporal distribution of foxes with sarcoptic mange and rabies (1967–1990). Figure S5. Foxes with sarcoptic mange analysed per year at the SRC. Figure S6. Spatiotemporal distribution of foxes with sarcoptic mange and/or rabies (1967–1990). Figure S7. Sarcoptic mange and rabies spread in Switzerland (1967–1992). [file 13071_2019_3762_MOESM3_ESM.pdf]
