# Supplementary material for: Spatiotemporal spread of sarcoptic mange in the red fox (Vulpes vulpes) in Switzerland over more than 60 years: lessons learnt from comparative analysis of multiple surveillance tools
Source: Parasit Vectors. 2019 Nov 5;12:521. doi: 10.1186/s13071-019-3762-7 (PMC6833187; doi:10.1186/s13071-019-3762-7)
Supplement: Supplementary file 4 — Additional file 4: Table S6. Answers to the questionnaire survey on sarcoptic mange in foxes per year (<1980–2017). Table S7. Answers to the questionnaire survey on sarcoptic mange in foxes per Swiss canton (<1980–2017). Figure S8. Changes in the occurrence of foxes with mange-like lesions in districts of surveillance per surface area. Figure S9. Temporal occurrence of mange-like lesions in foxes per number of cantons and districts of surveillance (questionnaire survey <1980–2017). Figure S10. Reliability categories of sarcoptic mange diagnosis in foxes (questionnaire survey 2001–2017). Figure S11. Trends in the occurrence of mange-like lesions in foxes in Switzerland (questionnaire survey 2001–2017). [file 13071_2019_3762_MOESM4_ESM.pdf]

Additional file 4

Questionnaire survey

Source: Centre for Fish and Wildlife Health (FIWI)

Table S6. Answers to the questionnaire survey on sarcoptic mange in foxes in Switzerland per year (<1980-2017)

Number of questionnaires returned, number of questionnaire reporting cases of mange-like lesions in red foxes (*Vulpes vulpes*), number of cases reported, type of observation related to the cases observed, and the tendency of the occurrence of mange-like lesions. *Abbreviations*: Lab, laboratory (confirmation through *S. scabiei* identification); MLL, mange-like lesions; Tot, total.

Years	Answers (n)		MLL cases (n)				Type of observation (n)				Tendency (n)		
	Tot	MLL	1-2	3-5	6-10	> 10	Live	Dead	Culled	Lab	Increasing	Stable	Decreasing
2017	227	171	34	51	33	53	129	121	154	6	39	103	43
2012	293	135	46	37	12	40	52	51	63	2	33	71	39
2011	360	181	53	52	29	47	86	87	102	12	48	77	43
2010	281	156	55	46	23	32	72	70	83	9	53	79	50
2009	267	120	49	29	10	32	58	64	65	10	48	87	32
2008	199	71	24	20	8	19	33	38	41	11	24	59	13
2007	228	69	29	15	11	14	34	39	50	8	24	83	23
2006	194	58	19	13	15	11	31	38	50	12	13	42	16
2005	234	58	12	17	9	20	38	39	40	32	17	50	24
2004	227	55	17	13	9	16	37	38	45	24	19	62	19
2003	466	61	16	17	7	16	36	32	47	6	25	78	27
2002	258	53	12	18	8	15	33	32	44	7	21	40	6
2001	164	47	12	16	12	7	27	30	32	8	10	35	10
1995-00	164	44	-	-	-	-	-	-	-	-	-	-	-
1990-94	164	17	-	-	-	-	-	-	-	-	-	-	-
1980-89	164	26	-	-	-	-	-	-	-	-	-	-	-
<1980	164	36	-	-	-	-	-	-	-	-	-	-	-

Table S7. Answers to the questionnaire survey on sarcoptic mange in foxes per Swiss canton (<1980-2017)

Number of questionnaires returned and number and percentage of questionnaire reporting cases of mange-like lesions. *Abbreviations*: AG, Aargau; AI, Appenzell Innerhoden; AE, Appenzell Ausserhoden: BE, Bern; BL, Basel Landschaft; BS, Basel-Stadt; FR, Fribourg; GE, Geneva; GL, Glarus; GR, Graubünden; JU, Jura; NE, Neuchatel; NW, Nidwalden; OW, Obwalden; SG, Sankt Gallen; SH, Schaffhausen; SO, Solothurn; SZ, Schwyz; TG, Thurgau, TI, Ticino; UR, Uri; VS, Wallis; ZG, Zug; ZH, Zürich; MLL, mange-like lesions.

Canton	<1980		1980-1989		1990-1994		1995-2000		2001		2002		2003		2004		2005		2006		2007		2008		2009		2010		2011		2012		2017		<1980-2017		
	**Tot**	**MLL**	**Tot**	**MLL**	**Tot**	**MLL**	**Tot**	**MLL**	**Tot**	**MLL**	**Tot**	**MLL**	**Tot**	**MLL**	**Tot**	**MLL**	**Tot**	**MLL**	**Tot**	**MLL**	**Tot**	**MLL**	**Tot**	**MLL**	**Tot**	**MLL**	**Tot**	**MLL**	**Tot**	**MLL**	**Tot**	**MLL**	**Tot**	**MLL**	**Tot**	**MLL**	**%**
AG	1	1	1	1	1	0	1	0	1	0	1	0	200	5	0	0	0	0	0	0	7	6	0	0	1	1	4	4	7	7	0	0	1	1	226	26	12%
AI	1	0	1	0	1	0	1	0	1	0	1	0	1	0	0	0	0	0	0	0	0	0	1	0	1	0	1	1	1	1	0	0	1	1	12	3	25%
AE	1	0	1	0	1	0	1	0	1	0	1	0	1	0	0	0	1	0	1	0	1	0	0	0	0	0	1	1	1	1	0	0	1	1	13	3	23%
BE	25	4	25	5	25	1	25	8	25	13	29	14	29	16	28	17	31	16	26	18	26	16	9	8	26	23	32	31	31	30	16	16	25	22	433	258	60%
BL	1	0	1	0	1	0	1	0	1	0	0	0	0	0	0	0	1	0	0	0	0	0	1	1	0	0	0	0	1	1	0	0	1	1	9	3	33%
BS	1	1	1	1	1	0	1	0	1	0	0	0	1	0	1	0	1	0	0	0	0	0	1	1	0	0	0	0	0	0	0	0	1	0	10	3	30%
FR	15	1	15	1	15	0	15	1	15	0	14	0	16	4	13	4	15	4	14	6	15	6	4	4	6	6	6	5	11	11	4	4	13	12	206	69	33%
GE	1	0	1	0	1	0	1	1	1	1	1	1	1	1	1	1	1	1	1	1	0	0	1	1	0	0	1	1	0	0	0	0	1	1	13	10	77%
GL	4	1	4	0	4	1	4	0	4	0	4	0	4	0	3	0	4	0	3	0	2	0	4	0	3	0	4	0	4	1	0	0	4	3	59	6	10%
GR	12	3	12	1	12	0	12	4	12	4	12	2	15	8	14	8	12	6	7	6	9	9	9	9	11	7	2	2	10	9	10	9	12	8	183	95	52%
JU	0	0	0	0	0	0	0	0	0	0	3	0	3	0	2	0	3	0	3	1	3	0	0	0	4	0	2	1	3	3	3	3	3	3	32	11	34%
LU	1	0	1	0	1	0	1	0	1	0	1	0	1	0	0	0	3	3	4	4	2	2	19	19	37	36	46	45	122	58	122	57	1	1	363	225	62%
NE	2	1	2	0	2	0	2	0	2	0	2	0	3	0	2	0	0	0	0	0	0	0	1	1	3	3	2	2	3	3	3	3	3	3	32	16	50%
NW	2	0	2	1	2	0	2	0	2	0	2	0	0	0	1	0	0	0	1	0	1	0	0	0	0	0	0	0	0	0	0	0	3	3	18	4	22%
OW	2	1	2	0	2	0	2	1	2	1	3	2	3	2	1	1	1	1	1	1	3	2	3	3	3	2	3	2	3	2	3	1	3	2	40	24	60%
SG	7	3	7	0	7	0	7	1	7	0	8	0	7	0	6	0	6	0	6	0	5	0	7	0	7	2	3	1	5	3	0	0	7	7	102	17	17%
SH	1	0	1	0	1	0	1	0	1	0	1	0	0	0	0	0	0	0	2	0	0	0	0	0	0	0	0	0	0	0	0	0	1	1	9	1	11%
SO	33	5	33	5	33	3	33	4	33	3	32	3	37	1	35	0	25	1	0	0	25	4	23	3	32	6	51	21	29	16	14	13	10	10	478	98	21%
SZ	5	0	5	0	5	0	5	1	5	0	5	1	4	0	5	1	6	0	6	0	6	0	0	0	2	0	1	0	4	1	4	1	3	1	71	6	8%
TG	1	0	1	0	1	0	1	0	1	0	90	5	89	2	72	2	76	2	82	2	81	2	84	2	85	8	82	18	86	13	83	7	82	52	997	115	12%
TI	5	0	5	0	5	0	5	5	5	3	5	3	5	3	5	4	5	3	5	3	5	5	5	4	0	0	5	3	0	0	5	3	5	5	75	44	59%
UR	10	2	10	0	10	0	10	2	10	2	11	2	11	0	11	1	12	1	9	1	10	3	11	3	11	1	11	2	10	1	9	3	8	3	174	27	16%
VD	5	2	5	0	5	0	5	1	5	1	4	1	7	1	1	0	5	3	4	3	5	2	2	2	7	7	0	0	9	9	6	6	9	9	84	47	56%
VS	27	10	27	11	27	12	27	15	27	19	26	19	27	18	24	16	24	17	17	12	20	12	13	10	26	18	23	16	18	11	10	9	27	19	390	244	63%
ZG	1	1	1	0	1	0	1	0	1	0	1	0	0	0	1	0	1	0	1	0	1	0	1	0	1	0	0	0	1	0	0	0	1	1	14	2	14%
ZH	0	0	0	0	0	0	0	0	0	0	1	0	1	0	1	0	1	0	1	0	1	0	0	0	1	0	1	0	1	0	1	0	1	1	11	1	9%
Total	**164**	**36**	**164**	**26**	**164**	**17**	**164**	**44**	**164**	**47**	**258**	**53**	**466**	**61**	**227**	**55**	**234**	**58**	**194**	**58**	**228**	**69**	**199**	**71**	**267**	**120**	**281**	**156**	**360**	**181**	**293**	**135**	**227**	**171**	**4054**	**1358**	**33%**

Figure S8. Changes in the occurrence of foxes with mange-like lesions in districts of surveillance per surface area

Percentages of the Swiss surface area covered by districts of surveillance in which red foxes (*Vulpes vulpes*) with and without mange-like lesions were reported in a yearly questionnaire survey from <1980 to 2017. *Abbreviation*: MLL, mange-like lesions.

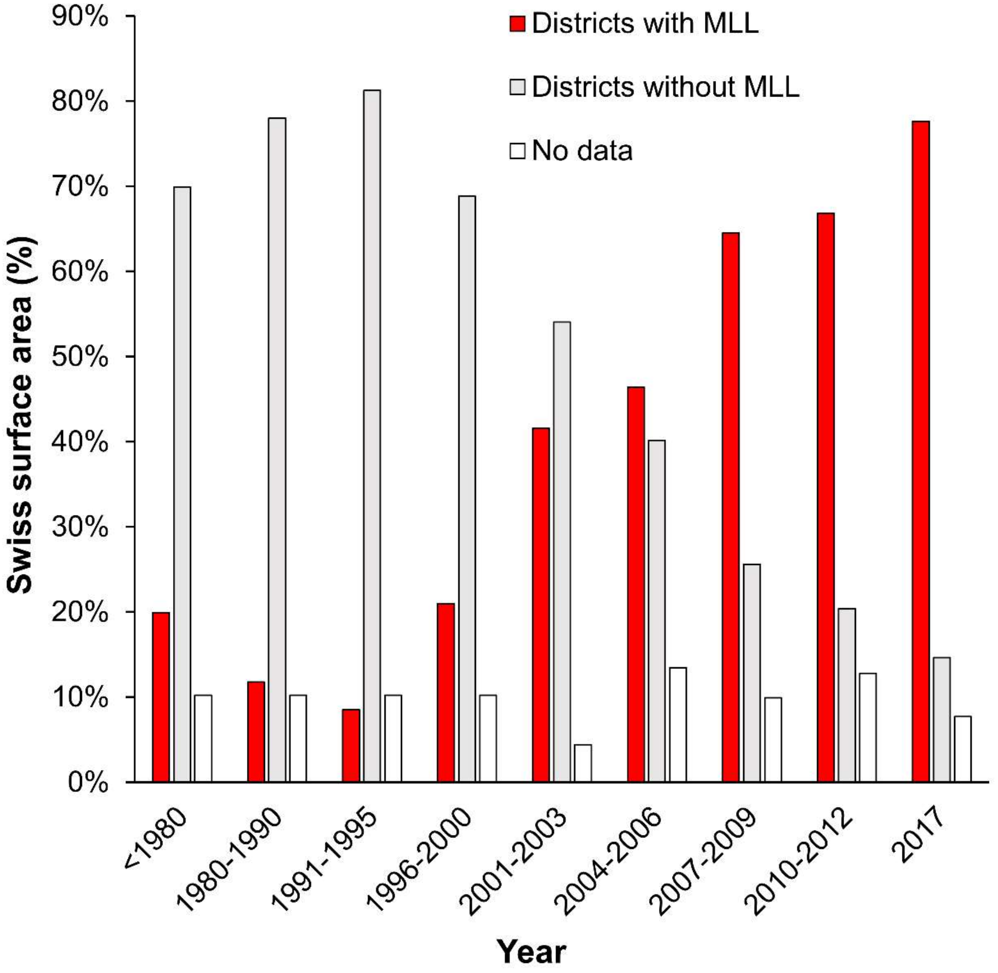

Figure S9. Temporal occurrence of mange-like lesions in foxes per number of cantons and districts of surveillance (questionnaire survey <1980-2017)

The percentage of Swiss cantons and districts of surveillance reporting red foxes (*Vulpes vulpes*) with mange-like lesions are indicated for nine periods. The lowest number of foxes with mange-like lesions reported yearly as a range is indicated as a mean for each period. *Abbreviation*: MLL, mange-like lesions.

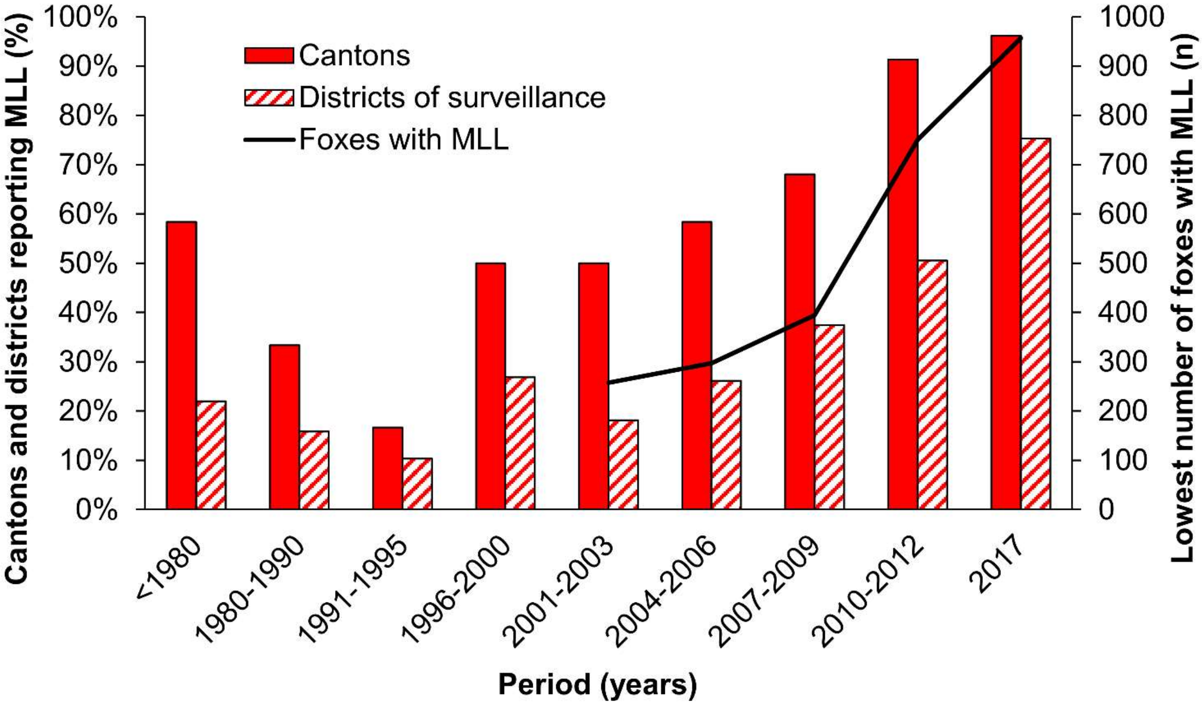

Figure S10. Reliability categories of sarcoptic mange diagnosis in foxes (questionnaire survey 2001-2017)

Three categories were defined according to the number and type of observations of mange-like lesions in red foxes (*Vulpes vulpes*). If mites were isolated in a laboratory in at least one fox of a district of surveillance, this district was placed in category 1 (confirmed diagnosis). If the diagnosis of sarcoptic mange remained presumptive, districts were placed either in category 2 or category 3: if three or more foxes with mange-like lesions were reported but mite presence was not confirmed, mange was considered as likely (category 2); if only one or two foxes with mange-like lesions were reported and mite presence was not confirmed, mange occurrence was considered uncertain (category 3). Pie charts show the percentages of the Swiss surface area according to the reliability categories of mange occurrence in districts of surveillance. In 2004-2005, more foxes with mange-like lesions were submitted for analysis due to a project on sarcoptic mange (Nimmervoll et al. 2013), explaining, at least partially, the higher number of districts with confirmed sarcoptic mange (category 1) during the period 2004-2006. *Abbreviation*: MLL, mange-like lesions.

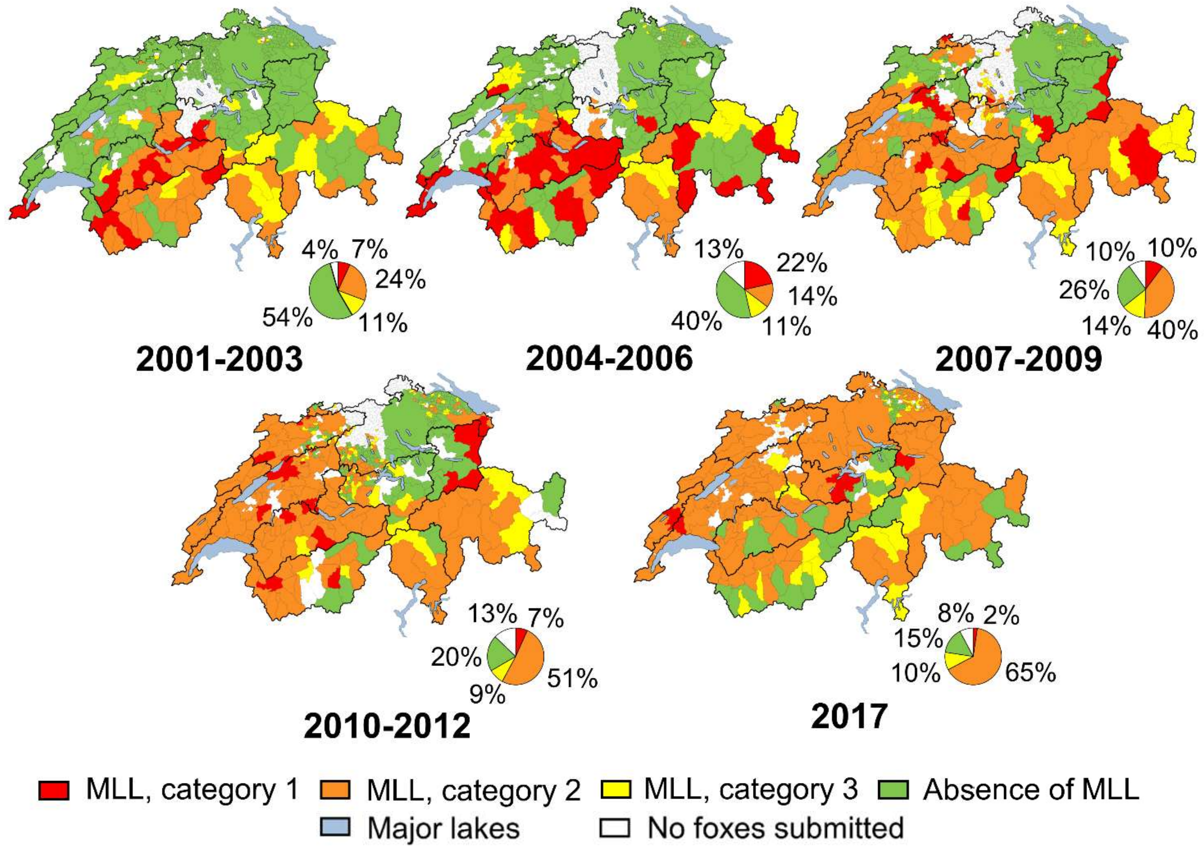

Figure S11. Trends in the occurrence of mange-like lesions in foxes in Switzerland (questionnaire survey 2001-2017)

Districts of surveillance of Switzerland are illustrated with different colours depending on the trends in the occurrence of mange-like lesions in red foxes (*Vulpes vulpes*), i.e. if the numbers of observed foxes with mange-like lesions were stable, increasing or decreasing. Pie charts represent the percentages of the surface area of districts of surveillance reporting stable, increasing or decreasing trends.

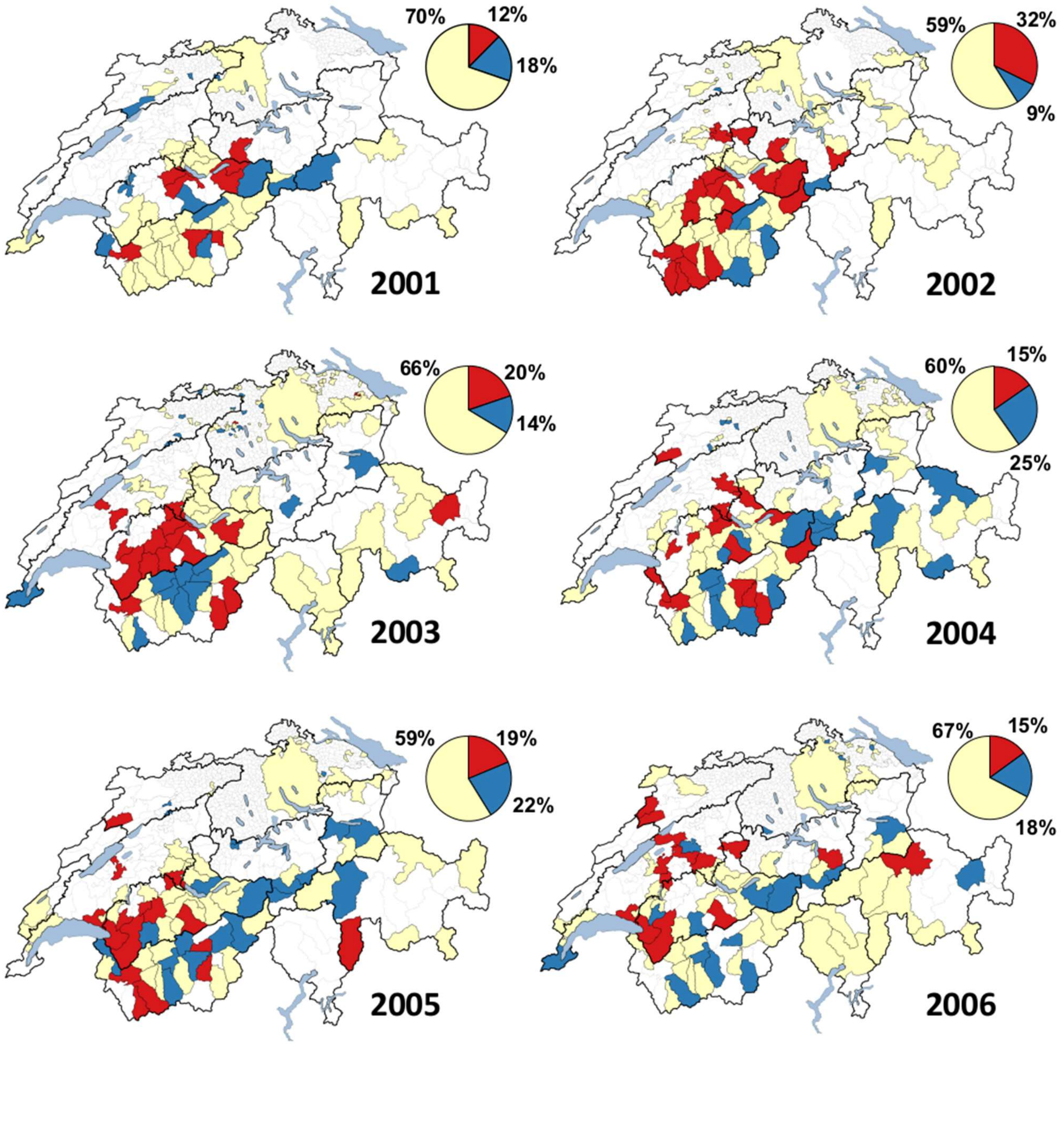

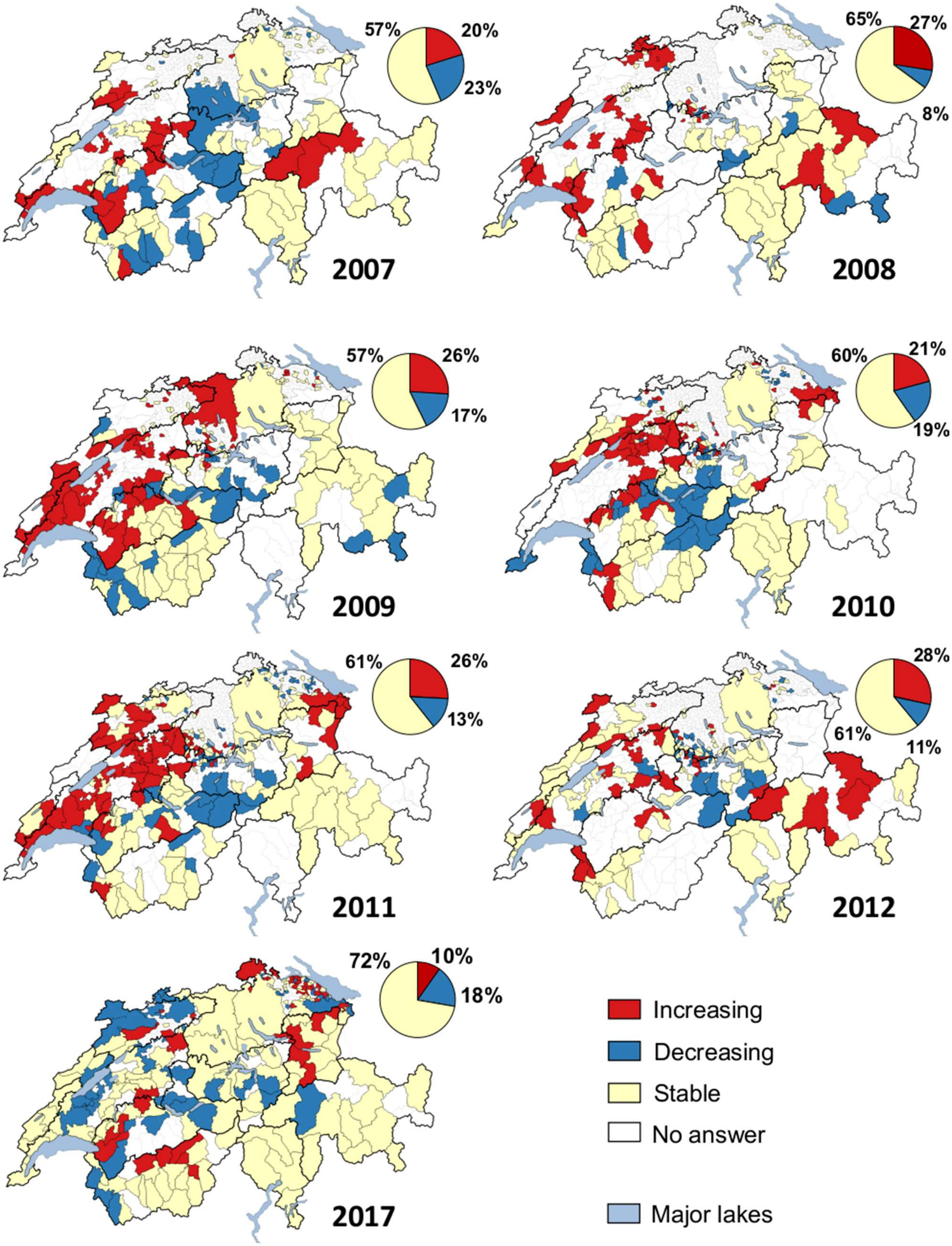
57%
20%
23%
2007
65%
27%
8%
2008
57%
26%
17%
2009
60%
21%
19%
2010
61%
26%
13%
2011
28%
61%
11%
2012
72%
10%
18%
2017
Increasing
Decreasing
Stable
No answer
Major lakes
