# Supplementary material for: Spatiotemporal spread of sarcoptic mange in the red fox (Vulpes vulpes) in Switzerland over more than 60 years: lessons learnt from comparative analysis of multiple surveillance tools
Source: Parasit Vectors. 2019 Nov 5;12:521. doi: 10.1186/s13071-019-3762-7 (PMC6833187; doi:10.1186/s13071-019-3762-7)
Supplement: Supplementary file 5 — Additional file 5: Table S8. Cantonal and national hunting statistics before, during and after the rabies epidemics. [file 13071_2019_3762_MOESM5_ESM.pdf]

Additional file 5: Table S8. Cantonal and national hunting statistics before, during and after the rabies epidemics

The number of foxes shot per km^2 (terrestrial surface below 2000 meters of altitude; Wandeler 1995) per year and per canton was calculated as an indicator of fox population density for four selected 4-year periods (before, 1946-1950; during, 1980-1984; and after the rabies epidemics, 1993-1997 and 2013-2017), the respective factor of increase (e.g. $HIPD_{80-84}/HIPD_{46-50}$) and rate of increase (e.g. $(HIPD_{80-84} – HIPD_{46-50})/(34years \times 100)$ were calculated according to Müller et al. 2001.

			Hunting bag[2] (n)				HIPD: shot fox / km^2 (yearly, mean)				Factor of increase			Rate of increase		
Cantons	Subregions	Surface[1] (km^2)	1946-50	1980-84	1993-97	2013-17	1946-50	1980-84	1993-97	2013-17	46-50/ 80-84	80-84/ 93-97	93-97/ 13-17	46-50/ 80-84	80-84/ 93-97	93-97/ 13-17
AG	EP, NJ	1375.29	3409	2851	11664	8592	0.62	0.52	2.12	1.56	0.84	4.09	0.74	-0.11	12.32	-2.79
AI	NEA	166.17	557	538	1632	1638	0.84	0.81	2.46	2.46	0.97	3.03	1.00	-0.17	12.66	0.05
AE	NEA	241.73	998	539	4158	2764	1.03	0.43	4.30	2.86	0.41	10.04	0.66	-0.10	29.79	-7.21
BE	WP, NWA, NJ	4933.48	12141	12491	39533	17653	0.62	0.63	2.00	0.89	1.03	3.16	0.45	1.35	10.54	-5.54
BL	EP, NJ	515.19	695	440	5927	2571	0.34	0.21	2.88	1.25	0.63	13.47	0.43	1.02	20.48	-8.14
BS	EP	35.53	0	49	113	33	0.00	0.34	0.80	0.23	NA	2.31	0.29	1.27	3.46	-2.81
FR	WP, NWA	1576.15	4461	1915	11060	5855	0.71	0.30	1.75	0.93	0.43	5.78	0.53	0.69	11.16	-4.13
GE	WP	242.02	104	77	591	59	0.11	0.08	0.61	0.06	0.74	7.68	0.10	-0.08	4.08	-2.75
GL	NEA	484.44	1073	1224	2862	1609	0.55	0.63	1.48	0.83	1.14	2.34	0.56	0.56	6.50	-3.23
GR	SEA	3014.64	9318	6435	17234	13721	0.77	0.53	1.43	1.14	0.69	2.68	0.80	-0.48	6.89	-1.46
JU	NJ	835.92	0	518	7780	3503	NA	0.15	2.33	1.05	NA	15.02	0.45	NA	16.71	-6.40
LU	EP, CA	1419.94	1986	3265	12483	9622	0.35	0.57	2.20	1.69	1.64	3.82	0.77	0.94	12.48	-2.52
NE	CJ	714.33	1654	787	3732	408	0.58	0.28	1.31	0.14	0.48	4.74	0.11	-0.83	7.93	-5.82
NW	CA	222.46	175	1016	1362	1138	0.20	1.14	1.53	1.28	5.81	1.34	0.84	3.11	2.99	-1.26
OW	CA	421.24	995	1202	1640	1583	0.59	0.71	0.97	0.94	1.21	1.36	0.97	0.57	2.00	-0.17
SG	EP, NEA	1793.08	5728	4192	17435	10710	0.80	0.58	2.43	1.49	0.73	4.16	0.61	-0.18	14.20	-4.69
SH	EA	295.56	1415	414	3355	2051	1.20	0.35	2.84	1.73	0.29	8.10	0.61	-2.65	19.14	-5.51
SO	NJ, WP	784.42	1484	1157	6928	4211	0.47	0.37	2.21	1.34	0.78	5.99	0.61	1.05	14.15	-4.33
SZ	CA	790.7	1767	1930	6337	5494	0.56	0.61	2.00	1.74	1.09	3.28	0.87	0.62	10.72	-1.33
TG	EP	856.08	2852	2222	9365	7233	0.83	0.65	2.73	2.11	0.78	4.21	0.77	-0.93	16.05	-3.11
TI	SA	2049.16	635	1487	2207	1016	0.31	0.18	0.27	0.12	0.59	1.48	0.46	-0.36	0.68	-0.73
UR	CA	524.36	839	1459	1972	1795	0.40	0.70	0.94	0.86	1.74	1.35	0.91	1.29	1.88	-0.42
VD	WP, SJ, NWA	2750.96	6129	2260	9529	4132	0.56	0.21	0.87	0.38	0.37	4.22	0.43	-0.18	5.08	-2.45
VS	SWA	2032.57	3915	7755	8231	8615	0.48	0.95	1.11	1.06	1.98	1.16	0.95	1.58	1.21	-0.26
ZG	EP, CA	205.96	558	416	2652	1503	0.68	0.50	3.22	1.82	0.75	6.38	0.57	0.08	20.88	-6.97
ZH	EA	1645.83	4601	4979	16448	10952	0.70	0.76	2.50	1.66	1.08	3.30	0.67	0.49	13.40	-4.17
Switzerland		29927.21	67489	61618	206230	128461	0.56	0.51	1.72	1.07	0.91	3.35	0.62	0.45	9.29	-3.25

Abbreviations: Cantons: AG, Aargau; AI: Appenzell Innerhoden; AE: Appenzell Ausserhoden: BE, Bern; BL, Basel Landschaft; BS: Basel-Stadt; FR, Fribourg; GE: Geneva; GL, Glarus; GR, Graubünden; JU, Jura; NE, Neuchâtel; NW, Nidwalden; OW, Obwalden; SG, Sankt Gallen; SH, Schaffhausen; SO, Solothurn; SZ,

Schwyz; TG, Thurgau, TI, Ticino; UR, Uri; VS, Wallis; ZG, Zug; ZH, Zürich; Biogeographical subregions: SJ: Southern Jura; CJ: Central Jura; Northern Jura; WP, Western Plateau; EP, Eastern Plateau; NWA; Northwestern Alps; CA, Central Alps; NEA, Northeastern Alps; SWA, Southwestern Alps; SA, Southern Alps; SEA, Southeastern Alps; HIPD, hunting indicator of population density.

Sources:

[1] Terrestrial surface below 2000 meters of altitude: Federal Statistical Office (FSO). Swisstopo: swissBoundaries3D 2018 (Kantone), swissAlti3d Stand 2012 (Höhe), Bundesamt für Statistik: Arealstatistik 04/09 mit teilweiser Revision (Wasser)

[2] Hunting bag: Federal Office of the Environment - federal hunting statistics (FOEN): uzh.ch/wild/static/jagdstatistik/
